# Comprehensive Analysis of Expression, Prognostic Value, and Immune Infiltration for Ubiquitination-Related FBXOs in Pancreatic Ductal Adenocarcinoma

**DOI:** 10.3389/fimmu.2021.774435

**Published:** 2022-01-03

**Authors:** Yalu Zhang, Qiaofei Liu, Ming Cui, Mengyi Wang, Surong Hua, Junyi Gao, Quan Liao

**Affiliations:** Department of General Surgery, State Key Laboratory of Complex Severe and Rare Diseases, Peking Union Medical College Hospital, Chinese Academy of Medical Science and Peking Union Medical College, Beijing, China

**Keywords:** FBXOs, pancreatic ductal adenocarcinoma, prognostic value, immune infiltration, bioinformatics analysis

## Abstract

Pancreatic ductal adenocarcinoma (PDAC) is one of the most refractory human malignancies. F-box only proteins (FBXO) are the core components of SKP1-cullin 1-F-box E3 ubiquitin ligase, which have been reported to play crucial roles in tumor initiation and progression *via* ubiquitination-mediated proteasomal degradation. However, the clinical implications and biological functions of FBXOs in PDAC have not been fully clarified. Herein we perform a comprehensive analysis for the clinical values and functional roles of FBXOs in PDAC using different public databases. We found that FBXO1 (CCNF), FBXO20 (LMO7), FBXO22, FBXO28, FBXO32, and FBXO45 (designated six-FBXOs) were robustly upregulated in PDAC tissues, which predicted an adverse prognosis of PDAC patients. There was a significant correlation between the expression levels of six-FBXOs and the clinicopathological features in PDAC. The transcriptional levels of six-FBXOs were subjected to the influence of promoter methylation levels. There were more than 40% genetic alterations and mutations of six-FBXOs, which affected the clinical outcome of PDAC patients. Furthermore, the expression of six-FBXOs was associated with immune infiltrations and activated status, including B cells, CD8^+^ T cells, CD4^+^ T cells, NK cells, macrophages, and dendritic cells. The functional prediction revealed that the six-FBXOs were involved in ubiquitination-related pathways and other vital signaling pathways, such as p53, PI3K/Akt, and Hippo pathway. Therefore, six-FBXOs are the promising prognostic biomarkers or potential targets for PDAC diagnosis and treatment.

## Introduction

According to the 2017 cancer statistics of the National Cancer Center of China, the incidence of pancreatic cancer ranked 11th among women and 7th among men, and the mortality rate ranked 6th among malignant tumors in China (1). Pancreatic ductal adenocarcinoma (PDAC) is the most common pathological type of pancreatic cancer, accounting for about 90% of pancreatic cancer (2). The clinical outcome of PDAC patients is often extremely poor, and the 5-year survival rate is only around 9% due to difficulties in early diagnosis, low success rate in surgical dissection, and chemotherapy resistance (3, 4). Therefore, it is necessary to find diagnostic or therapeutic targets of PDAC to improve the clinical outcomes of patients.

The occurrence and development of PDAC are responsible for multi-factor and multi-step processes, such as the activation/inactivation of oncogenes/tumor suppressor genes, tumor stem cell, gene mutation, epigenetic modification, and post-translational regulation as well as tumor immune microenvironment (5–8). Among them, ubiquitination-mediated degradation of target protein with high selectivity is a key pathway for post-translational modification, which plays a significant role in oncogenesis and pathological mechanism (9). E3 ubiquitin ligase is a vital component of the ubiquitination cascade that binds directly to substrates by controlling mutual specificity, which is regarded as a promising anticancer drug target (10). F-box only (FBXO) proteins are the substrate-recognizing subunits of SKP1-cullin 1-F-box (SCF) E3 ligase. An increasing number of studies have revealed that FBXO family members, as important molecular regulators, are implicated in cell apoptosis, angiogenesis, epithelial–mesenchymal transition (EMT), and multiple important signaling pathways, like p53, NF-κB, PI3K/AKT, and Hippo signaling pathway, in a variety of tumors (11); for example, FBXO1, also known as cyclin F (CCNF), participates in the formation of SCF ubiquitin ligase complex and is involved in tuning centrosome duplication, DNA repair, and genome stability (12). FBXO20, also known as LIM (Lin11, Isl-1, and Mec-3) domain 7 (LMO7), belongs to PDZ and the LIM domain-containing protein family. It has been reported that LMO7 played a role in the regulation of cell adhesion, mitosis, and cancer metastasis and progression, including breast cancer, lung adenocarcinoma, and pancreatic cancer (13–16). Clinically, some FBXO family members were closely linked to the overall survival (OS) and disease-free survival (DFS) of patients with breast cancer (17). However, the clinical significance and biological role of FBXOs in PDAC are still unclear as yet and remain to be elucidated.

Among FBXO1–50, FBXO12 is also called FBXO35, FBXO17 is also called FBXO26, FBXO14 is also called FBXO31, and FBXO19 is absent. In the present study, we mainly focused on six FBXO family members with limited studies in the field of PDAC research, including FBXO1, FBXO20, FBXO22, FBXO28, FBXO32, and FBXO45 (designated six-FBXOs in the following text). We compared the different expression levels of the six ubiquitination-related FBXOs between PDAC tissues and paracarcinoma tissues or normal pancreatic tissues. Furthermore, using diverse public databases, we comprehensively analyzed their correlation with clinicopathologic characteristics, promoter methylation in epigenetic regulation, fluorescence localization, gene mutation, immune infiltration, and prognostic values as well as the functional enrichment analysis and the prediction of Kyoto Encyclopedia of Genes and Genomes (KEGG) pathway. Finally, we validated the expression levels of six-FBXOs in five different PDAC cell lines compared with human normal pancreas cells.

## Materials and Methods

### GEPIA

GEPIA is an interactive online web tool for investigators to explore diverse functional modules based on TCGA and GTEx databases (18). Using the GEPIA database, we analyzed the gene expression profile of FBXO family members across 33 tumor samples and paired normal tissues, including pancreatic adenocarcinoma (PAAD), and performed the pathological stage plot of the FBXOs as well as evaluated the prognostic values of FBXOs in PDAC patients. “Median” was selected as the “Group Cutoff”.

### Oncomine and UALCAN

Oncomine is a public database that contains substantial tumor microarray datasets (19). Log2-transformed form was utilized to represent the transcriptional levels of FBXOs. Fold change >2 and *p*-value <0.05 were selected as the inclusion criteria. UALCAN is a user-friendly database for the analysis of gene expression and clinical parameters from thirty-one different types of tumors, including PDAC (20). In this study, PDAC staging, grading, TP53 mutation, and methylation data were obtained from the UALCAN database.

### GEO

GEO database includes a large number of high-throughput sequencing data, including PDAC datasets. We used five GEO datasets to compare the expression levels of FBXOs in PDAC tissues and normal pancreatic tissues (GSE16515 and GSE62165) or para-carcinoma tissues (GSE62452, GSE28735, and GSE15471). Besides these, five GEO datasets were used to analyze correlations between the expression levels of FBXOs and the clinicopathologic characteristics of PDAC. GSE21501 was used for the analysis of tumor size (T1–T2 *vs*. T3–T4) and lymphatic metastasis (N0 *vs*. N1); GSE62165, GSE62452, GSE19650, and GSE51971 were used for the analysis of tumor location (head *vs*. body/tail), differentiation degree (G1–G2 *vs*. G3–G4), different pancreatic precancerous lesions, and cell stemness, respectively.

### The Human Protein Atlas

The Human Protein Atlas (HPA) database is an integrated and accessible data mining platform containing a substantial distribution of information of human protein from more than twenty kinds of cancer in cellular and histopathological levels. The immunohistochemical (IHC) staining images of FBXO family members and the confocal images of their cellular localization were acquired from the HPA database.

### Kaplan–Meier Plotter

The Kaplan–Meier plotter is an interactive and public online database for analyzing the survival of twenty-one tumor types based on substantial RNA-seq and next-generation sequencing (21). The prognostic values of FBXO family members and their prognostic subgroup analysis in gender, mutation burden, and immune infiltration were evaluated by the Kaplan–Meier plotter database. Hazard ratio (HR) and 95% confidence interval (CI) were calculated automatically according to “Auto select best cutoff”.

### SurvExpress and LinkedOmics

SurvExpress is a bioinformatics web server for gene expression and clinical data in tumors (22). The prognosis of FBXO family members and the transcriptional levels in low- and high-risk groups were assessed by using this database. “PAAD-TCGA-Pancreatic adenocarcinoma” (*n* = 176) was selected. The PDAC cohorts were divided into low- and high-risk groups based on the prognostic index to compare the expression levels of FBXOs. LinkedOmics is an available web portal for users to analyze multi-omics data within and across 32 cancer types (23). This database was used to evaluate the prognosis of promoter methylation of the FBXOs and top 200 co-expressed genes with individual FBXOs in PDAC patients.

### Immune Infiltration Analysis of FBXOs

TIMER is a user-friendly web server for the systematic and comprehensive analysis of immune infiltration across various tumor types *via* inputting function-specific parameters (24). The immune infiltration of FBXOs in PDAC tissues was estimated using TIMER database (Spearman correlation). Furthermore, TISIDB is a public portal for tumor and immune system interaction as well as integration of numerous heterogeneous data types (25). We used this database to perform a further detailed analysis of immune infiltration of FBXOs in PDAC based on high-throughput data.

### cBioPortal Database

The cBioPortal database is an accessible online resource to interactively explore and visualize multidimensional genomics data and clinical profiles in diverse cancer samples (26). Using cBioPortal database, we analyzed the genetic alteration, mutation, and related prognosis of FBXOs in PDAC and identified the top 200 co-expressed genes of FBXOs. The “Pancreatic adenocarcinoma (TCGA, PanCancer Atlas)” (184 samples) was selected. In terms of “Select Genomic Profiles”, the “Mutation”, “Structural Variant”, “Putative copy-number alterations from GISTIC”, “mRNA Expression”, and “Protein expression z-scores (RPPA)” were selected. Besides these, “Complete sample (168)” was selected as the option of “Select Patient/Case Set”.

### DAVID

DAVID is a bioinformatic analytic tool for the functional enrichment of genes derived from high-throughput genomic experiments, such as functional classification and annotation, to acquire an in-depth understanding of the biological function of gene lists (27). Functional enrichment and prediction of FBXOs were performed using gene ontology (GO) and KEGG pathway analysis from DAVID. GO functional enrichment consists of biological process (BP), cellular component (CC), and molecular function (MF).

### Cell Culture

Human immortal pancreatic epithelial cell line HPDE6 and PDAC cell lines (Panc-1, AsPC-1, SW1990, T3M4, and CAPAC-1) were purchased from the National Infrastructure of Cell Line Resource (Beijing, China). HPDE6, Panc-1, and T3M4 were cultured in DMEM medium (HyClone); AsPC-1, SW1990, and CFPAC-1 were cultured in RPMI-1640, Leibovitz’s L-15, and IMDM medium (Coning), respectively. All media were supplemented with 10% fetal bovine serum (Gibco) and 1% penicillin–streptomycin (Sigma), and cells were incubated in a humidified atmosphere at 37°C and 5% CO_2_.

### RNA Extraction and Quantitative PCR

Total RNA was isolated from exponentially divided cell lines using Trizol Reagent (RNAiso Plus, #9109, Takara) and then concentrated with trichloromethane, isopropanol, and ethanol according to standard protocols. The RNA was reverse-transcribed with PrimeScript™ RT reagent Kit (#RR037A, Takara). Real-time qPCR was administrated in three duplicates for each sample with TB Green^®^ Premix Ex Taq™ (#RR420A, Takara) on the QuantStudio3 apparatus (ThermoFisher, USA). GAPDH was used for standard normalization, and the data were analyzed using the 2^−ΔΔCT^ method. The primer sequences were summarized in **Supplementary Table S1**.

### Statistical Analysis

Statistical analysis and graphs were performed and plotted by GraphPad Prism 6.0 (Lajolla, CA, USA). Comparisons between the two groups were conducted using a two‐tailed Student’s *t*-test. For comparisons of three or more groups, one-way ANOVA with *post-hoc* Dunnett test or Tukey’s test was utilized. Statistical significance was indicated as *p*-value <0.05.

## Results

### Aberrant Transcriptional Levels of FBXOs in Patients With PDAC

**Figure 1:**
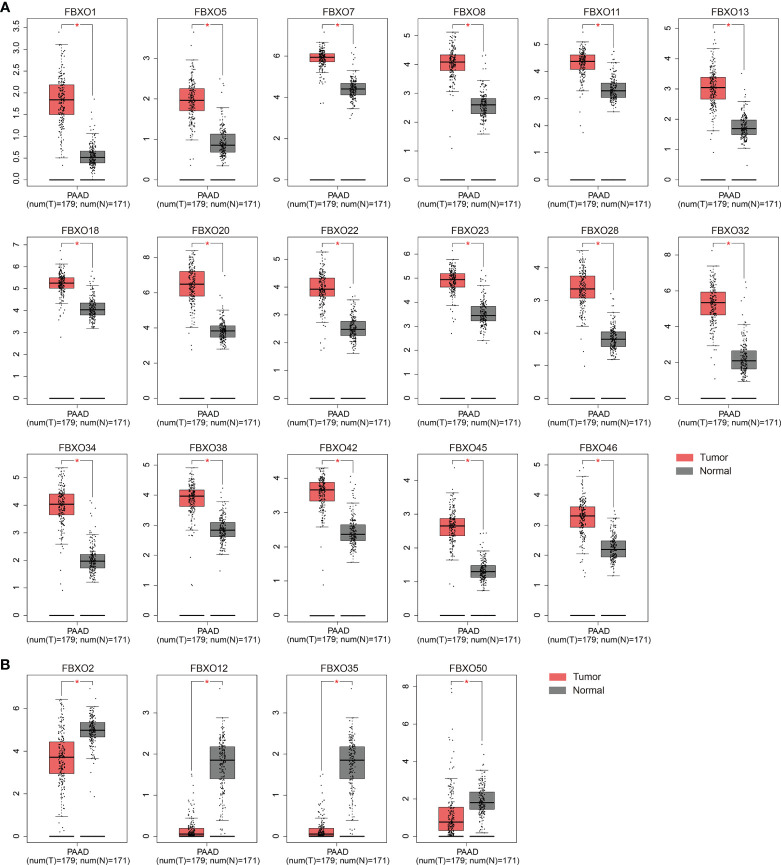
Using GEPIA database, FBXO family members that are aberrantly expressed in pancreatic ductal adenocarcinoma (PDAC) tissues (*n* = 179) *versus* normal pancreatic tissues (*n* = 171). **(A)** FBXO family members that are highly expressed in PDAC tissues. **(B)** FBXO family members that are lowly expressed in PDAC tissues. *P < 0.05.

**Figure 2:**
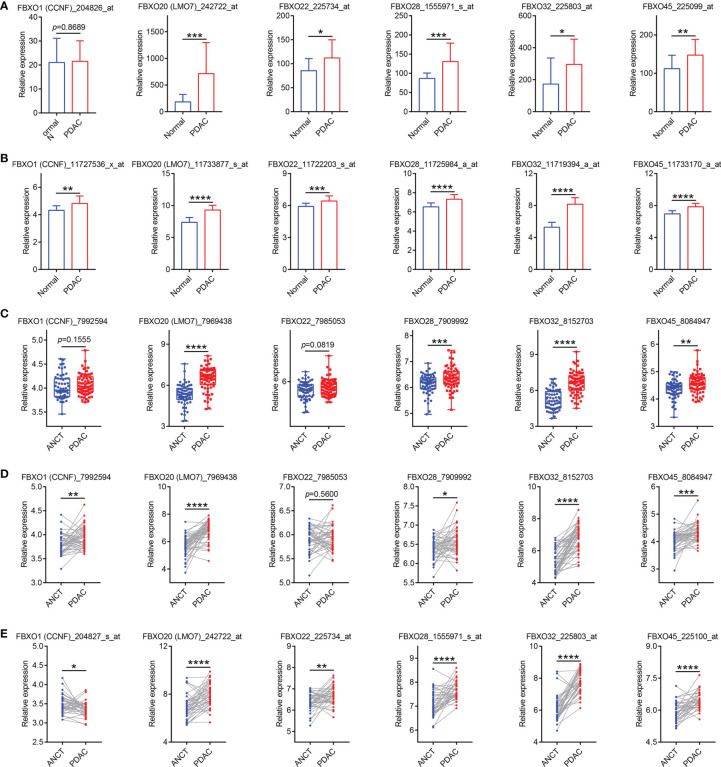
Five GEO datasets were collected to validate the abnormality in the expression patterns of six-FBXOs in pancreatic ductal adenocarcinoma (PDAC). **(A)** The mRNA levels of six-FBXOs *via* the comparison between PDAC (*n* = 36) and normal pancreatic tissues (*n* = 16) in GSE16515 dataset. **(B)** The expression levels of six-FBXOs in PDAC tissues (*n* = 118) compared with normal tissues (*n* = 13) in GSE62165 dataset. **(C)** The expression levels of six-FBXOs in PDAC tissues (*n* = 69) *versus* adjacent non-cancerous tissues (ANCT) (*n* = 61) in GSE62452 dataset. **(D)** The transcriptional levels of six-FBXOs in PDAC tissues compared with ANCT (*N* = 45 pairs) in GSE28735 dataset. **(E)** The expression levels of six-FBXOs in PDAC tissues compared with ANCT (*N* = 39 pairs) in GSE15471 dataset. The code behind the gene is the probe name in different GEO datasets. *P < 0.05, **P < 0.01, ***P < 0.001, ****P < 0.0001.

To investigate the expression profiles of FBXO family members in PDAC, the GEPIA database, an online analysis tool based on TCGA and GTEx datasets, was used to compare the difference between the mRNA expression levels of FBXOs in PDAC tissues (*N* = 179) and normal pancreatic tissues (*N* = 171). A total of twenty-one aberrantly expressed genes were identified within the human PDAC tissues. Of them, FBXO1, FBXO5, FBXO7, FBXO8, FBXO11, FBXO13, FBXO18, FBXO20, FBXO22, FBXO23, FBXO28, FBXO32, FBXO34, FBXO38, FBXO42, FBXO45, and FBXO46 were upregulated, while FBXO2, FBXO12, FBXO35, and FBXO50 were downregulated in PDAC tissues (**Figure 1**). No abnormal expression was found in other FBXO members (**Supplementary Figure S1**). By consulting the previously published literature, there are extremely limited reports related to FBXO1, FBXO20, FBXO22, FBXO28, FBXO32, and FBXO45 in PDAC, and we therefore further explored the six genes. Furthermore, the transcriptional levels of six-FBXOs were explored in 32 other types of tumor. We found that six-FBXOs were also abnormally expressed in the other multiple tumors (**Supplementary Figure S2**). Next, Oncomine databases were also used to investigate the expression of six-FBXOs in PDAC tissues *versus* normal pancreatic tissues. The results confirmed that the mRNA expression levels of FBXO20, FBXO32, and FBXO45 were strongly upregulated in patients with PDAC in different datasets (**Supplementary Figures S3A–D** and **Table 1**). In Buchholz’s data, FBXO32 exhibited a higher expression in pancreatic duct intraepithelial neoplasia (PanIN) than in normal pancreatic tissues (**Supplementary Figure 3C**). Moreover, five GEO datasets were collected to verify the abnormality in the expression patterns of six-FBXOs found above *via* a comparison between PDAC and normal pancreatic tissues (GSE16515 and GSE62165) or adjacent non-cancerous tissues (ANCT) (GSE62452, GSE28735, and GSE15471). As shown in **Figure 2**, the overall results confirmed that the expression levels of six-FBXOs were robustly elevated, although FBXO1 and FBXO22 did not show statistical differences in two of the datasets (**Figures 2A–E**).

**Table 1:** The significant changes of FBXO transcription levels between PDAC tissues and normal pancreatic tissues (Oncomine).

Genes	Source	Type of pancreatic cancer *versus* normal pancreatic tissue	Platform	*t*-test	Fold change	*p-*value
FBXO20 (LMO7)	Pei pancreas	Pancreatic carcinoma *vs*. normal	Human Genome U133 Plus 2.0 Array	7.962	3.535	1.56E-10
	Iacobuzio-Donahue pancreas 2	Pancreatic adenocarcinoma *vs*. normal	NA	6.309	4.305	1.04E-05
	Segara pancreas	Pancreatic carcinoma *vs*. normal	Human Genome U133A Array	3.061	1.481	0.005
FBXO32	Badea pancreas	Pancreatic ductal adenocarcinoma *vs*. normal	Human Genome U133 Plus 2.0 Array	10.146	3.427	6.17E-16
	Iacobuzio-Donahue pancreas 2	Pancreatic adenocarcinoma *vs*. normal	NA	4.855	2.640	1.10E-04
	Grutzmann pancreas	Pancreatic ductal adenocarcinoma epithelia *vs*. normal	Human Genome U133B Array	2.588	2.246	0.009
	Buchholz pancreas	Pancreatic intraepithelial neoplasia *vs*. normal	NA	3.191	1.339	0.004
	Buchholz pancreas	Pancreatic ductal adenocarcinoma *vs*. normal	NA	4.784	1.543	3.24E-04
	Pei pancreas	Pancreatic carcinoma *vs*. normal	Human Genome U133 Plus 2.0 Array	3.007	2.657	0.004
FBXO45	Grutzmann pancreas	Pancreatic ductal adenocarcinoma epithelia *vs*. normal	Human Genome U133B Array	2.530	2.173	0.010
	Badea pancreas	Pancreatic ductal adenocarcinoma *vs*. normal	Human Genome U133 Plus 2.0 Array	5.406	1.502	3.62E-07
	Iacobuzio-Donahue pancreas 2	Pancreatic adenocarcinoma *vs*. normal	NA	2.054	1.850	0.042
	Pei pancreas	Pancreatic carcinoma *vs*. normal	Human Genome U133 Plus 2.0 Array	3.020	1.348	0.003

NA, not available.

### The Correlation Between Six-FBXO Expression and the Clinicopathological Features of PDAC Patients

**Figure 3:**
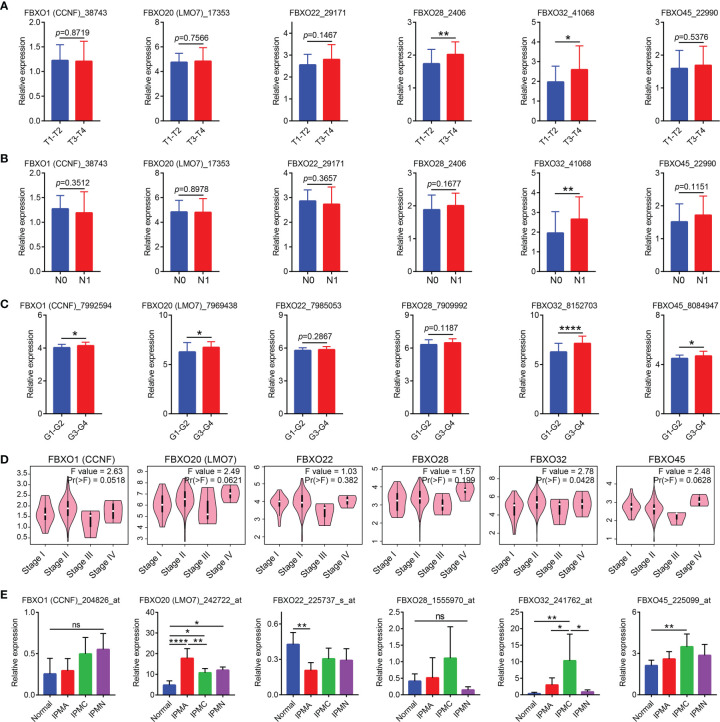
Correlation between six-FBXO expression and the clinicopathological characteristics of pancreatic ductal adenocarcinoma (PDAC) patients in GEO datasets and GEPIA. **(A)** The expression levels of six-FBXOs *via* the comparison between T1–T2 (*n* = 18) and T3–T4 (*n* = 80) in GSE21501 dataset. **(B)** The expression levels of six-FBXOs in PDAC patients with/without lymphatic metastasis (N1/N0, 73 *vs*. 28) in GSE21501 dataset. **(C)** The mRNA levels of six-FBXOs in different differentiation degrees of PDAC (G1–G2 *vs*. G3–G4, 37 *vs*. 31) using GSE62452 dataset. **(D)** The expression levels of six-FBXOs in different pathological stages using GEPIA. **(E)** The transcriptional levels of six-FBXOs in different pancreatic precancerous lesions compared with normal pancreatic tissues using GSE19650 dataset. Normal (*n* = 7), IPMA (*n* = 6), IPMC (*n* = 6), and IPMN (*n* = 3). The code behind the gene is the probe name in different GEO datasets. *P < 0.05, **P < 0.01 ****P < 0.0001. n.s., not significant difference.

Then, we evaluated the clinical significance of six-FBXOs in PDAC by using three GEO datasets (GSE62165, GSE21501, and GSE62452), GEPIA, and UALCAN databases. The data from GSE62165 showed that none of the six-FBXOs was associated with tumor location (*N* = 118) (**Supplementary Figure S4A**), while GSE21501 displayed that FBXO28 and FBXO32 were tightly linked to tumor size (*N* = 98); FBXO32 was also related to lymphatic metastasis (*N* = 101) (**Figures 3A, B** **)**. GSE62452 demonstrated that the higher expression levels of FBXO1, FBXO20, FBXO32, and FBXO45 were correlated with poorer tumor differentiation (*N* = 68), apart from FBXO22 and FBXO38 (**Figure 3C**). Besides this, the GEPIA database manifested that, among six-FBXOs, FBXO32 was associated with tumor staging (*N* = 179); FBXO1, FBXO20, and FBXO45 had a related trend with tumor staging, whereas no correlation with tumor staging was observed for FBXO22 and FBXO28 (**Figure 3D**). It was worth mentioning that FBXO family members FBXO8, FBXO13, and FBXO34 were linked to tumor staging, and FBXO50 had an associated trend with tumor staging (**Supplementary Figure S4B**).

The results from the UALCAN database further confirmed the GSE21501 data that FBXO32 was closely relevant in the lymphatic metastasis of PDAC as well as showed that FBOX20 expression was relatively lower in PDAC patients with diabetes, compared with PDAC patients without diabetes (**Supplementary Figure S4C**). Furthermore, the UALCAN database displayed a more detailed analysis regarding tumor differentiation and staging based on TCGA samples. The data from the UALCAN database verified that the expression levels of six-FBXOs were strongly relevant in tumor differentiation, and patients who were in worse differentiation had the higher expression of six-FBXOs on the whole (**Supplementary Figure S5A**). Further validating the results of the GEPIA database, FBXO32 was related to the tumor stage of PDAC patients. It also indicated that the mRNA expression levels of FBXO20 and FBXO45 were closely related to the tumor stage of PDAC patients (**Supplementary Figure S5B**), which meant that patients who were in more advanced tumor staging tended to express higher transcripts of FBXO20, FBXO32, and FBXO45. TP53 mutation plays a crucial role in the initiation, formation, and maintenance of PDAC (28). Therefore, we also explored the effect of P53 mutation on the expression of six-FBXOs. The data showed that FBXO1, FBXO20, and FBXO45 exhibited higher expression levels in the TP53-mutant group than in the TP53-nonmutant group, while this kind of difference was not observed for FBXO22, FBXO28, and FBXO32 (**Supplementary Figure S5C**).

Pancreatic carcinogenesis is a gradual and multi-step process from precancerous lesions (8). We compared the expression of six-FBXOs in different precancerous lesions, including intraductal papillary-mucinous neoplasm (IPMN), intraductal papillary-mucinous carcinoma (IPMC), and intraductal papillary-mucinous adenoma (IPMA) by using the GSE19650 dataset. The results indicated that FBXO20 was highly expressed in these three kinds of precancerous lesions (IPMA, IPMC, and IPMN) in comparison to normal pancreatic tissues, and FBXO20 had the highest expression level among these three pathological types, while FBXO22 was downregulated in IPMA compared with corresponding normal tissues (**Figure 3E**). FBXO32 and FBXO45 were significantly enhanced in IPMC, and FBXO32 exhibited the highest expression level among the other pathological types (**Figure 3E**). Taken together, these results demonstrated that FBXO20, FBXO22, FBXO32, and FBXO45 may act as markers of precancerous lesions and have the value of early diagnosis of pancreatic cancer.

### The Correlation Between Promoter Methylation Levels of Six-FBXOs and Clinicopathological Characteristics

Promoter methylation is a critical epigenetic modification to regulate gene expression in human cancers (29). Therefore, we next investigated the promoter methylation levels of six-FBXOs. The results illustrated that FBXO1, FBXO20, and FBXO32 had lower methylation levels in primary tumors than in normal pancreatic tissues, while FBXO45 had a higher methylation level in PDAC (**Supplementary Figure S6A**). In six-FBXOs, there were considerable differences in the promoter methylation level among divergent differentiated degrees or clinical stages. The methylation levels of FBXO1, FBXO20, FBXO32, and FBXO45 were associated with tumor differentiation (**Supplementary Figure S6B**). The methylation levels of FBXO1, FBXO20, FBXO22, FBXO32, and FBXO45 were correlated with clinical stages (**Supplementary Figure S7A**). In particular, FBXO1 exhibited a robust negative correlation between promoter methylation levels and differentiated degree or clinical stages. In addition, FBXO1, FBXO20, and FBXO45 had a lower methylation degree in the TP53-mutant group than in the TP53-nonmutant group, while FBXO22, FBXO28, and FBXO32 had no significant correlation between methylation level and TP53-mutant (**Supplementary Figure S7B**).

### The Protein Expression, Cell Lines, Cellular Localization, and Cell Stemness of Six-FBXOs in PDAC

**Figure 4:**
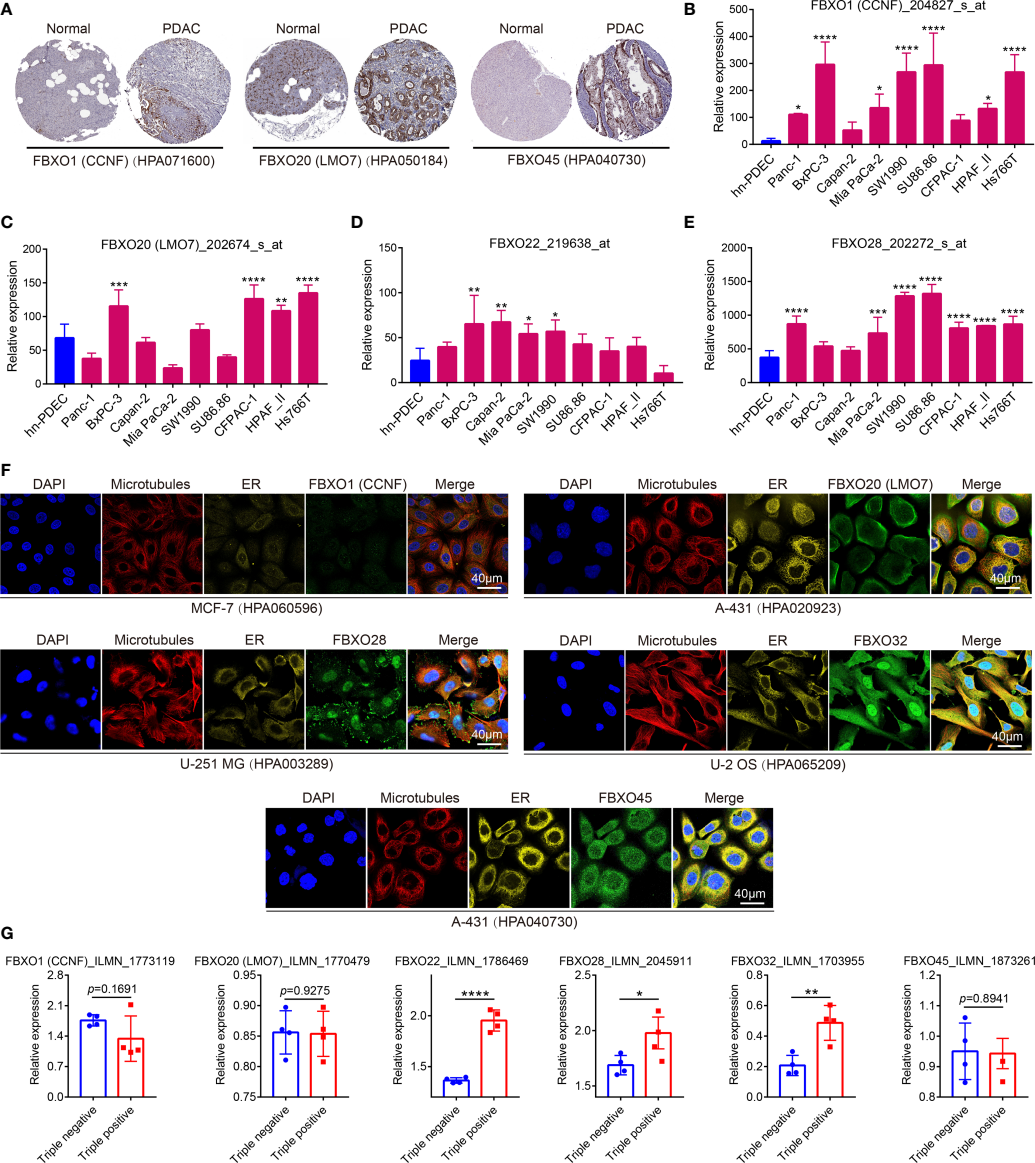
The protein expression, cell lines, cellular localization, and cell stemness of six-FBXOs in pancreatic ductal adenocarcinoma (PDAC). **(A)** The protein expression of FBXO1, 20, and 45 in PDAC and normal tissues using immunohistochemistry (IHC) in Human Protein Atlas (HPA) database. The IHC images of FBXO22, 28, and 32 were absent. The code behind the gene is the related primary antibody in HPA. **(B–E)** The expression levels of FBXO1, 20, 22, and 28 in human normal pancreatic ductal epithelial cells (hn-PDEC) and multiple PDAC cell lines using GSE45757 dataset. The code behind the gene is the related probe name. FBXO32 and FBXO45 were not available. **(F)** The confocal images of cellular localization of six-FBXOs in different types of cells using HPA database. The code behind the cell line is the related primary antibody in HPA. ER, endoplasmic reticulum. **(G)** The expression levels of six-FBXOs in triple-positive group (*n* = 4) and triple-negative group (*n* = 4), which were classified by three key cancer stem cell markers, CD44, CD133, and EpCAM, using GSE51971 dataset. The code behind the gene is the related probe name. *P < 0.05, **P < 0.01, ***P < 0.001, ****P < 0.0001.

Next, we probed into the protein levels of six-FBXO expression between PDAC tissues and normal pancreatic tissues from the HPA database. The IHC staining of six-FBXOs revealed that the protein expression levels of FBXO1, FBXO20, and FBXO45 were markedly increased compared with the corresponding normal pancreatic tissues (**Figure 4A**), which corroborated the aforesaid result. The IHC data of FBXO22, FBXO28, and FBXO32 were pending cancer tissue analysis in the HPA database. Besides this, GSE45757 was used to evaluate the six-FBXO expression in human normal pancreatic ductal epithelial cells (hn-PDEC) and PDAC cell lines, including Panc-1, BxPC-3, Capan-2, Mia PaCa-2, SW1990, SU86.86, CFPAC-1, HPAF_II, and Hs766T. The data demonstrated that FBXO1, FBXO20, FBXO22, and FBXO28 were strongly upregulated in multiple PDAC cell lines relative to hn-PDEC (**Figures 4B–E**). The expression levels of FBXO32 and FBXO45 in cell lines were not available in the GSE45757 dataset. The cellular localization of gene expression often determines its corresponding significant function, so we explored the subcellular localization of six-FBXOs by using confocal images of the HPA database. The results warranted that FBXO1 was detected in the centrosome, FBXO20 was detected in actin filaments and cytosol, FBXO28 was detected in the nucleoplasm and focal adhesion sites, FBXO32 was detected in the nucleoplasm and cytosol, and FBXO45 was detected in the cytosol (**Figure 4F**). The confocal result of FBXO22 was absent in the HPA database. More importantly, we also explored the cell stemness of six-FBXOs in PDAC by using the GSE51971 dataset, whose data were classified into triple-positive group and triple-negative group according to whether these expressed the three important cancer stem cell markers, CD44, CD133, and EpCAM. The results showed that the expression levels of FBXO22, FBXO28, and FBXO32 were potently enhanced in the triple-negative group (**Figure 4G**). Overall, these results suggested that six-FBXOs are likely to play a vital role in PDAC initiation and progression.

### The Prognostic Analysis of Six-FBXOs in PDAC Patients

**Figure 5:**
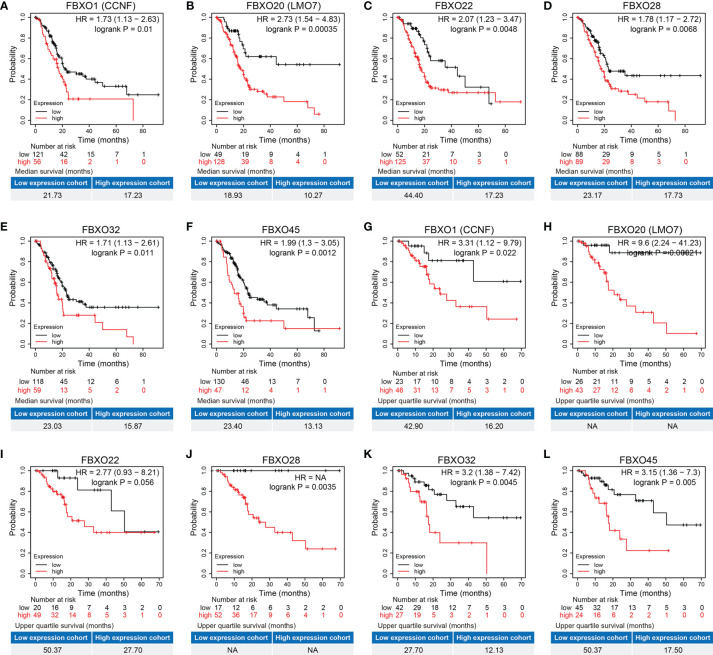
The prognostic analysis of the six-FBXOs in pancreatic ductal adenocarcinoma (PDAC) using Kaplan–Meier plotter. **(A–F)** The overall survival of the six-FBXOs in PDAC patients (*N* = 177). **(G–L)** The disease-free survival of the six-FBXOs in PDAC patients (*N* = 69). “Auto-select best cutoff” was selected. NA, not available.

In order to investigate the prognostic values, we explore the prognostic data of six-FBXOs from three primary public databases: GEPAI, Kaplan–Meier plotter, and SurvExpress. The data from GEPAI displayed that the high levels of six-FBXOs were tightly linked to a decreased probability of OS and DFS in PDAC (89 *vs*. 89) (**Supplementary Figure S8** and **Table 2**). The Kaplan–Meier plotter further substantiated the aforesaid prognostic results (**Figure 5** and **Table 2**). Specifically, FBXO20 had the highest HR in OS and DFS at 2.73 and 9.6, respectively, which meant that the FBXO20 high-expression group had 2.73 times and 9.6 times the risk of PDAC-related death and relapse, respectively, *versus* the FBXO20 low-expression group. The median OS time of the low- and high-expression groups was 18.93 and 10.27 months, respectively. More detailed prognostic information was shown in **Figure 5** and **Table 2**. Additionally, the SurvExpress online web tool also confirmed that six-FBXOs were the negative prognosis factors of PDAC patients (**Supplementary Figures S9**, **S10A**). The respective six-FBXO expression levels of PDAC patients in the SurvExpress datasets were divided into low- and high-risk groups (88 *vs*. 88) according to the prognostic index. The data from SurvExpress revealed that six-FBXOs had higher expression levels in the high-risk group than in the low-risk group (**Supplementary Figure S10B**). The LinkedOmics databases demonstrated that the hypermethylation group of FBXO1, FBXO20, and FBXO32 indicated a better OS than the hypomethylation group, while no significant correlation with OS was observed for FBXO22, FBXO28, and FBXO45 (**Supplementary Figure S11**). Collectively, these results in this section outlined the important prognostic values of six-FBXOs, which may act as effective prognostic markers for PDAC patients.

**Table 2:** Prognostic analysis of the six-FBXOs in GEPIA and Kaplan–Meier plotter.

Genes	Expression	GEPIA				Kaplan–Meier plotter			
		OS		DFS		OS		RFS	
		HR (high)	*p*-value	HR (high)	*p*-value	HR (95% CI)	*p*-value	HR (95% CI)	*p*-value
FBXO1 (CCNF)	Upregulated	1.6	0.024	1.4	0.098	1.73 (1.13–2.63)	0.0102	3.31 (1.12–9.79)	0.0224
FBXO20 (LMO7)	Upregulated	1.7	0.012	1.6	0.033	2.73 (1.54–4.83)	0.0004	9.60 (2.24–41.23)	0.0002
FBXO22	Upregulated	2.0	0.0014	1.6	0.037	2.07 (1.23–3.47)	0.0048	2.77 (0.93–8.21)	0.0561
FBXO28	Upregulated	1.6	0.026	1.6	0.026	1.78 (1.17–2.72)	0.0068	NA	0.0035
FBXO32	Upregulated	1.5	0.048	1.6	0.051	1.71 (1.13–2.61)	0.0110	3.20 (1.38–7.42)	0.0045
FBXO45	Upregulated	1.7	0.013	1.7	0.015	1.99 (1.30–3.05)	0.0012	3.15 (1.36–7.30)	0.0050

OS, overall survival; DFS, disease-free survival; RFS, relapse-free survival; HR, hazard ratio; CI, confidence interval; NA, not available.

### The Six-FBXO Expression and Immune Infiltration Level in PDAC

**Figure 6:**
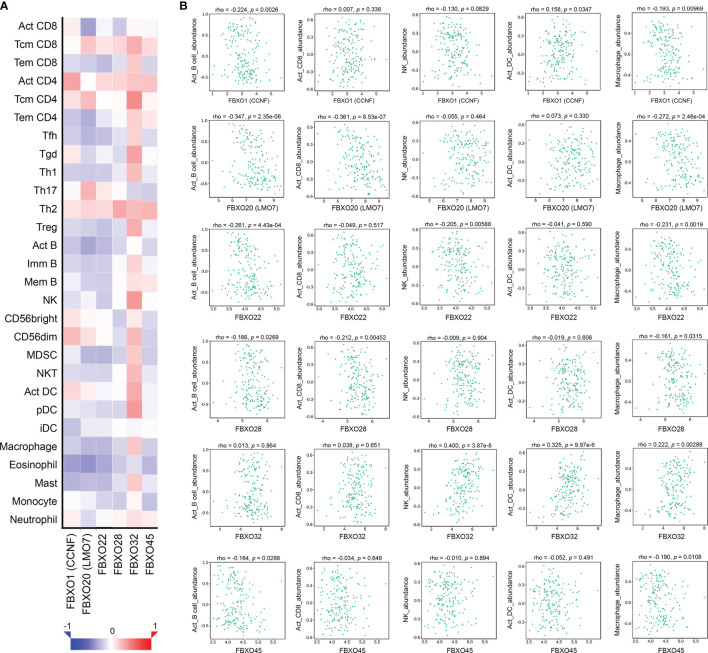
Association of six-FBXO expression with immune infiltration level in pancreatic ductal adenocarcinoma (PDAC) (TISIDB). **(A)** Heat map of immune infiltrate correlation for six-FBXOs. **(B)** Scatter plot of the correlation between the expression level of six-FBXOs and immune cell infiltration, including Act_B cells, Act_CD8^+^ T cells, NK cells, Act_DC, macrophages (Spearman correlation, *N* = 179). Act, activated; NK, natural killer cells; DC, dendritic cells.

Then, we analyzed the relationship between the expression of six-FBXOs and the immune infiltration levels in PDAC using the TIMER online tool. The data revealed that the expression levels of FBXO1, FBXO22, FBXO28, and FBXO45 were associated with B cell infiltration; FBXO20, FBXO22, FBXO28, FBXO32, and FBXO45 were correlated with CD8^+^ T cell infiltration; FBXO20 and FBXO45 were inversely related to CD4^+^ T cell infiltration; FBXO22, FBXO28, FBXO32, and FBXO45 were linked to macrophage and neutrophil cell infiltration; FBXO1, FBXO22, FBXO28, FBXO32, and FBXO45 were relevant in dendritic cell infiltration (**Supplementary Figure S12**). However, it was contradictory that these negative prognostic genes were positively associated with immune infiltration, especially CD8^+^ T cells, which often predicted a good clinical outcome. We speculated that this was related to whether immune cells were activated and contained different subgroups. Therefore, the TISIDB databases were utilized to perform a more detailed immune infiltration analysis of six-FBXOs in PDAC among subgroups with different functions. The results from TISIDB showed that, on the whole, six-FBXOs had a negative association with various immune infiltration, especially activated B cell, activated CD8^+^ T cell, and macrophage infiltration (**Figures 6A, B** **)**, indicating the expression of six-FBXOs might be associated with a suppressive tumor immune microenvironment in PDAC.

To determine whether these six-FBXOs affected the prognosis of immune subgroup in PDAC patients, the Kaplan–Meier plotter was used to evaluate it between the immune cell-enriched group and the immune cell-decreased group. The data from the Kaplan–Meier plotter illustrated that FBXO1, FBXO20, FBXO22, FBXO28, and FBXO32 were the negative prognosis factors in the B cell-decreased group and the macrophage-decreased group, while FBXO45 was the negative prognosis factor in the B cell-enriched group and the macrophage-enriched group. FBXO20 and FBXO45 were the negative prognosis factors in the CD8^+^ T cell-decreased group, while FBXO1, FBXO22, FBXO28, and FBXO32 were the negative prognosis factors in the CD8^+^ T cell-enriched group. All six-FBXOs were the negative prognosis factors in the regulatory T cell-enriched group (**Supplementary Figure S13**). On the whole, the six-FBXOs tended to be the negative prognosis factors in the immunosuppressed state of PDAC. Furthermore, we also detected the prognostic analysis of subgroups in different gender or mutation burden. The results showed that FBXO1 and FBXO32 were the negative prognosis factors in the female group, whereas FBXO20, FBXO22, FBXO28, and FBXO45 were the negative prognosis factors in the male group. FBXO1, FBXO20, FBXO22, FBXO28, and FBXO32 were the negative prognosis factors in the low-mutation-burden group, and FBXO45 was the negative prognosis factor in the high-nutation-burden group (**Supplementary Figure S13**). For the subgroup analysis of DFS, the data are shown in **Supplementary Figure S14**. The HR, 95% CI, and *p*-value for the OS and DFS of six-FBXOs in different subgroups are summarized in **Supplementary Table S2**.

### The Genetic Alteration and Mutation of Six-FBXOs in PDAC

**Figure 7:**
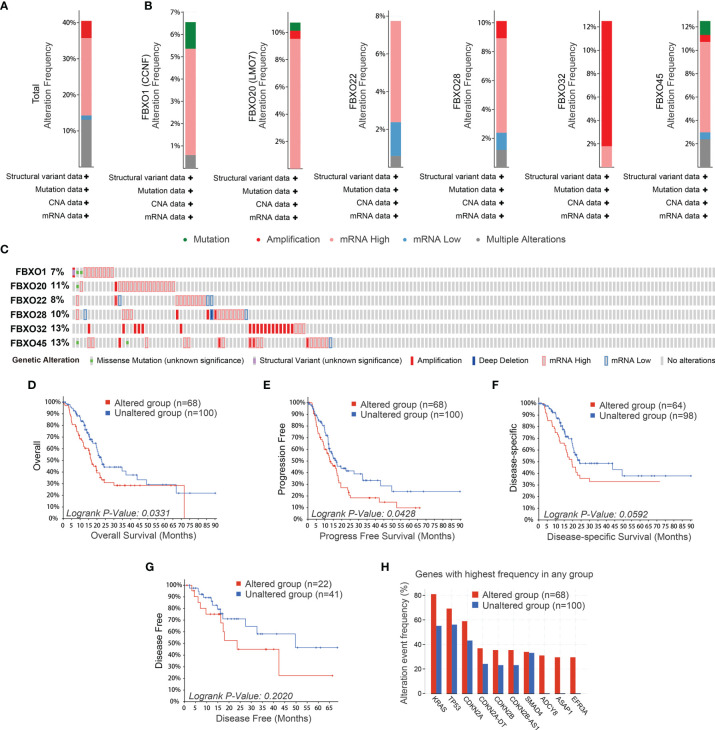
The genetic alteration and mutation of six-FBXOs in PDAC using cBioPortal database. **(A)** Total alteration frequency of the six-FBXOs in PDAC. **(B, C)** Individual alteration frequency of six-FBXOs in PDAC. **(D–G)** The prognostic analysis of the genetic alteration of the six-FBXOs in PDAC, including overall (*p* = 0.0331), progression-free (*p* = 0.0428), disease-specific (*p* = 0.0592), and disease-free survival (*p* = 0.2020). **(H)** Alteration event frequency of common mutant genes between altered group (*n* = 68) and unaltered group (*n* = 100).

We investigated the genetic alterations of the six-FBXO and the mutation in the cBioPortal database for pancreatic adenocarcinoma (TCGA and PanCancer Atlas). The results indicated that the genetic alteration of six-FBXOs in PDAC patients accounted for more than 40% (**Figure 7A**). Genetic alterations account for about 10% of each gene on average (**Figures 7B, C** **)**, including mutation, amplification, mRNA high, mRNA low, and multiple alterations. For the progression of the genetic alterations of six-FBXOs, the data manifested that the altered group had a worse OS (*p* = 0.0331) and progression-free survival (*p* = 0.0428) than the unaltered group (**Figures 7D, E** and **Supplementary Table S3**). There is also such a trend for disease-specific survival in PDAC (*p* = 0.0592), but no statistical difference for DFS of two groups (*p* = 0.2020) (**Figures 7F, G** and **Supplementary Table S3**). Besides these, the altered group always had the higher typical PDAC gene mutations than the unaltered group, such as KRAS, TP53, CDKN2A, *etc.* (**Figure 7H**). Next, we analyzed the difference in sample-level enrichments between the altered group and the unaltered group. Using *p <*0.05 and log ratio >2 as the screening conditions, 1,194 genes were obtained in the altered group. The top 20 differently enriched genes are listed in **Supplementary Table S4**, such as ADCY8, ASAP1, EFR3A, KCNQ3, and PCAT1, which had been reported to play roles in tumor onset and progression (30–32).

### Prediction of Functions and Pathways for Six-FBXOs in PDAC

**Figure 8:**
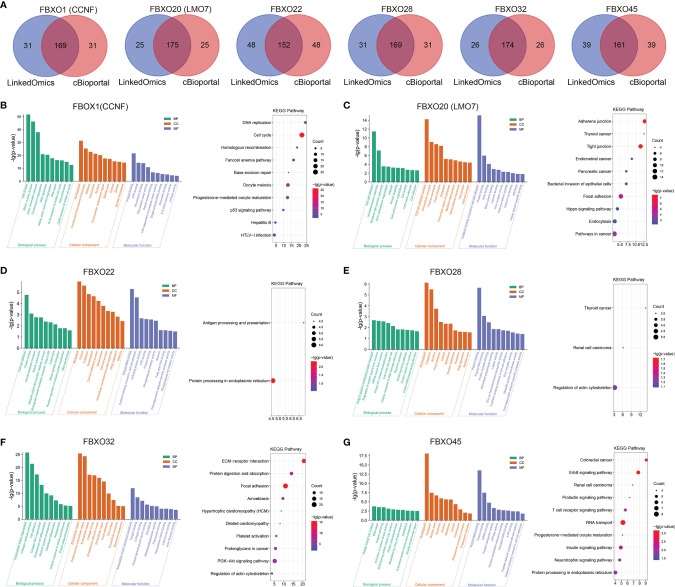
Comprehensive bioinformatics analysis of six-FBXOs and individual co-expressed genes in pancreatic ductal adenocarcinoma. **(A)** Intersection of genes positively co-expressed in the top 200 of the LinkedOmics and the cBioPortal database (Spearman correlation). **(B–G)** The Gene Ontology enrichment analysis and Kyoto Encyclopedia of Genes and Genomes pathway prediction of six-FBXOs were performed. BP, biological processes; CC, cellular components; MF, molecular function.

We screened the top 200 positively co-expressed genes using the LinkedOmics database and the cBioPortal database, respectively, and obtained the intersected genes (**Figure 8A**). These shared gene sets were used to predict the functions and pathways of six-FBXOs by performing the GO and KEGG enrichment analysis. The GO enrichment analysis contains three points: BP, CC, and MF.

For FBXO1, BP terms indicated cell division, mitotic nuclear division, DNA replication, DNA repair, cell cycle, and cell proliferation. CC terms were implicated in nucleoplasm and condensed chromosome kinetochore, midbody. MF terms were involved in protein binding, ATP binding, and protein kinase binding. The KEGG pathways showed that FBXO1 was related to cell cycle, DNA replication, and p53 signaling pathway (**Figure 8B**). For FBXO20, BP terms were implicated in cell–cell adhesion, hemidesmosome assembly, actin cytoskeleton organization, and negative regulation of extrinsic apoptotic signaling pathway. CC terms were involved in cell–cell adherens junction, extracellular exosome, plasma membrane, and focal adhesion. MF terms contained cadherin binding involved in cell–cell adhesion, protein binding, and actin binding. The KEGG pathways indicated that FBXO20 was related to tight junction, pathways in cancer, and Hippo signaling pathway (**Figure 8C**). For FBXO22, BP terms were implicated in protein folding, vesicle-mediated transport, and ubiquitin-dependent protein catabolic process. CC terms were involved in membrane, cytosol, and cytoplasm. MF terms contained poly(A) RNA binding, cadherin binding involved in cell–cell adhesion, and unfolded protein binding. The KEGG pathways indicated that FBXO22 was related to protein processing in the endoplasmic reticulum, antigen processing, and presentation (**Figure 8D**). For FBXO28, BP terms were implicated in Golgi to plasma membrane protein transport, regulation of protein export from nucleus, protein K11-linked deubiquitination, and EGFR signaling pathway. CC terms were involved in nucleoplasm, cytoplasm, and nucleus. MF terms contained protein complex binding, K63-linked polyubiquitin binding, and ubiquitin-protein transferase activity. The KEGG pathways indicated that FBXO28 was related to the regulation of actin cytoskeleton (**Figure 8E**). For FBXO32, BP terms were implicated in extracellular matrix organization, collagen catabolic process, cell differentiation, and angiogenesis. CC terms were involved in extracellular matrix, proteinaceous extracellular matrix, and extracellular space. MF terms contained extracellular matrix structural constituent and integrin binding. The KEGG pathways indicated that FBXO32 was related to ECM–receptor interaction, PI3K-Akt signaling pathway, proteoglycans in cancer, and regulation of actin cytoskeleton (**Figure 8F**). For FBXO45, BP terms were implicated in protein import into the nucleus, DNA repair, and activation of protein kinase activity. CC terms were involved in nucleoplasm, cytoplasm, and nuclear pore. MF terms contained poly(A) RNA binding, protein binding, and mRNA binding. The KEGG pathways indicated that FBXO45 was related to RNA transport, ErbB signaling pathway, insulin signaling pathway, and T cell receptor signaling pathway (**Figure 8G**).

### Validation of the Expression of Six-FBXOs in PDAC Cell Lines

**Figure 9:**
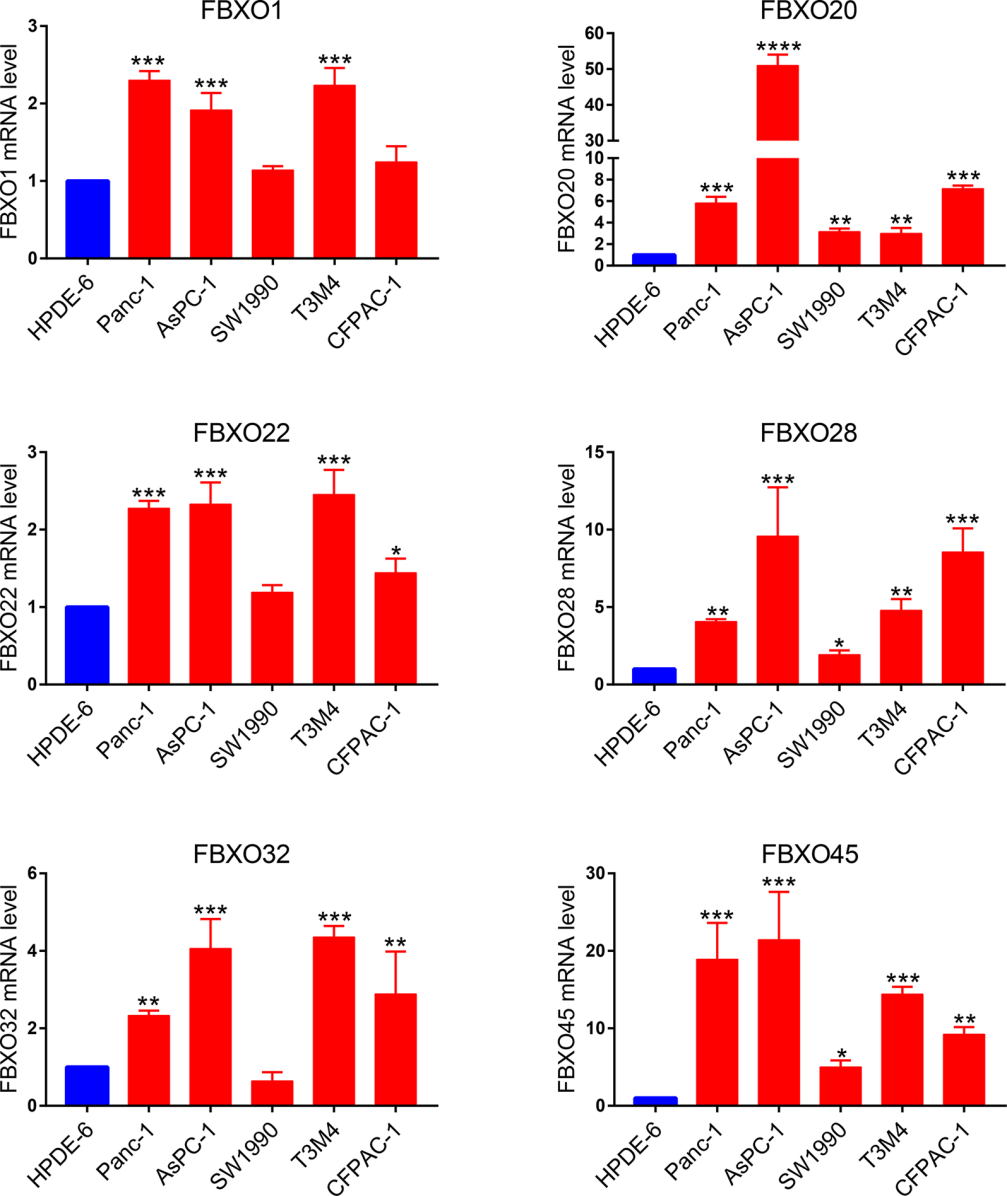
The expression levels of six-FBXOs in human immortalized normal pancreatic epithelial cells HPDE6 and different pancreatic ductal adenocarcinoma cell lines were verified using real-time qPCR. *P < 0.05, **P < 0.01, ***P < 0.001, ****P < 0.0001.

To further validate the findings of the bioinformatics analysis, we used qPCR to confirm the transcriptional levels of six-FBXOs in five pancreatic cancer cell lines, respectively, that is, Panc-1, AsPC-1, SW1990, T3M4, and CFPAC-1, compared with human immortal pancreatic epithelial cell line HPDE6. The results revealed that, overall, the expression levels of six-FBXOs are markedly elevated in multiple PDAC cell lines (**Figure 9**), in concordance with the above-mentioned data, and it substantiated our bioinformatics analysis to some extent, although further *in vitro* and *in vivo* experiments were needed to support it.

## Discussion

Herein we comprehensively analyzed the clinical significance, function, and prognostic value of FBXO family genes, especially the six-FBXOs (FBXO1, FBXO20, FBXO22, FBXO28, FBXO32, and FBXO45). Firstly, we found the multiple FBXO family members to be aberrantly expressed in PDAC and identified six-FBXOs that were inversely associated with the OS and DFS of PDAC patients. Notably, although some differences of GEO data were not large, all of them were statistically significant and mutually verified using databases from different sources, which could confirm our findings on the six-FBXOs. Furthermore, their correlations between expression and clinicopathologic characteristics, as well as the promoter methylation levels, were further analyzed by using GEO, GEPIA, and UALCAN databases. Next, we investigated their protein expression, cell lines, cellular localization, and cell stemness. Using different databases, the related immune infiltration, genetic alteration, and mutation were evaluated in PDAC tissues. Finally, we predicted their functions and pathways through positively co-expressed genes using GO and KEGG enrichment analysis. Although some FBXO family members have been reported to be involved in oncogenesis and tumor advancement (11, 17, 33), comprehensive bioinformatics analyses of PDAC have yet to be investigated. The present study is the first time to analyze the transcription levels, clinicopathologic characteristics, promoter methylation, mutation, immune infiltration, and prognostic values of six FBXO family members (FBXO1, FBXO20, FBXO22, FBXO28, FBXO32, and FBXO45) in PDAC. We hope that these findings will help to replenish the available knowledge between the FBXO family and PDAC and contribute to improve the accuracy of prognostic prediction in PDAC patients as well as provide potential effective targets for the diagnosis and treatment of PDAC.

### FBXO1

FBXO1 (CCNF) had been reported to be downregulated in hepatocellular carcinoma and was related to poor differentiation and adverse clinical outcome (34). In breast cancer, the overexpression of FBXO1 could suppress tumor progression, indicating that it has the role of a tumor suppressor (35). Intriguingly, FBXO1 mRNA was highly expressed in primary breast cancer tissues, but its protein level was strikingly reduced (35), hinting that FBXO1 was subjected to post-transcriptional modifications and protein degradation. Of note is the fact that FBXO1 was recently reported to be involved in the modification of the ubiquitination-proteasome system (36). FBXO1 was negatively regulated by the E3 ligase (FZR1) and the co-regulator of E3 ligase (FBXL8), which were known to play an oncogenic role in breast cancer, and FBXO1 could inhibit the expression of ribonucleotide reductase M2 (RRM2), a pro-tumorigenic protein (35). In contrast, FBXO1 was overexpressed in ovarian cancer tissues and facilitated the cell growth and invasion of ovarian cancer (37). Herein we found that FBXO1 was upregulated in PDAC tissues and the majority of PDAC cell lines as well as associated with the unfortunate prognosis of PDAC patients based on different public databases. These findings have been validated by a recently published study regarding the protein expression of FBXO1 in PDAC *via* IHC (38). Furthermore, our data showed that FBXO1 expression was linked to tumor differentiation, pathological grading, promoter methylation, P53 mutation, and immune infiltration in PDAC.

### FBXO20

FBXO20 (LMO7) was downregulated in lung adenocarcinoma and relevant in tumor size, nodal involvement, and pathological stage as well as with a poor prognosis (15). The depletion of FBXO20 resulted in an increase of susceptibility for spontaneous lung cancer in the murine model (39). In contrast, FBXO20 could facilitate the migration ability of breast cancer cells in a cell-specific manner *via* modulating Rho-MRTF-SRF signaling (14). Liu et al. verified that the expression levels of FBXO20 mRNA and protein were increased in mouse and human pancreatic cancer tissues (16). The results of Liu were consistent with our findings in multiple public databases to some extent. FBXO20 acted an oncogenic role to promote metastasis and progression. In terms of proliferation, FBXO20 exhibited dual roles, that is, cell cycle acceleration and apoptosis inhibition (16). In addition, our data showed that a higher expression of FBXO20 tended to result in poorer pathological grading and worse tumor differentiation and predict an adverse prognosis in PDAC patients. FBXO20 was significantly elevated in different pancreatic precancerous lesions, including IPMA, IPMC, and IPMN. Among them, IPMA had the highest expression levels of FBXO20. These data indicated that FBXO20 possessed the potential to be a sensitive indicator of precancerous lesions, which is important for the early diagnosis of PDAC. Besides this, FBXO20 was tightly correlated with diabetic status, promoter methylation, P53 mutation, and immune infiltration in PDAC. The functional analysis and KEGG pathways revealed that FBXO20 might be involved in apoptosis-related signaling pathway and Hippo signaling pathway, which deserve to be further investigated.

### FBXO22

Accumulating evidence suggested that FBXO22 exerts its oncogenic functions through mediating the ubiquitination and degradation of substrates in many human malignancies (40). However, the roles it plays in different tumors is inconsistent in physiological and pathological processes. In renal cell carcinoma, FBXO22 restrained cancer metastasis and progression by suppressing VEGF-induced angiogenesis and MMP-9-induced invasion and migration (41). On the contrary, FBXO22 facilitated tumorigenesis and progression *via* regulating the ubiquitination and degradation of p21 in hepatocellular carcinoma (42), of LKB1 in lung cancer, and of nuclear PTEN in colorectal cancer (43). Interestingly, FBXO22 exhibits a paradoxical dual role of pro-tumorigenic and anti-metastatic function in breast cancer progression (44). The functional role of FBXO22 in PDAC has not yet been reported. Herein we explored that FBXO22 was upregulated in PDAC and associated with cell stemness, poor pathological grading, and worse clinical outcomes. Notably, FBXO22 expression was closely correlated with the infiltration of a variety of immune cells, including B cells, CD8^+^ T cells, CD4^+^ T cells, NK cells, macrophages, neutrophils, and dendritic cells, implicating that it might be involved in the reprogramming of the tumor immune microenvironment in PDAC. Similarly, our functional analysis suggested that FBXO22 was related to the ubiquitin-dependent protein catabolic process, protein processing in the endoplasmic reticulum, antigen processing, and presentation, which allows our data and the published literatures to be mutually corroborated.

### FBXO28

SCF (Skp1/Cul1/F-box) ubiquitin ligase serves as a key modulator of cell homeostasis *via* regulating downstream a variety of critical proteins for ubiquitylation (45). The IHC analysis displayed that FBXO28 and its phosphorylation are strong and predicted a poor prognosis in breast cancer (46). The CDK1/2-driven activation of the E3 ubiquitin ligase SCF^FBXO28^ accelerated MYC-dependent transcription by non-proteolytic ubiquitylation and promoted transformation and tumorigenesis (46). Fagerholm et al. showed that FBXO28 had a correlation with survival and treatment outcome using interaction analysis of cis-eQTL variants in breast cancer (47). Herein we found that FBXO28 had a high level in the majority of PDAC cell lines and tissues, which often predicted an adverse clinical survival for PDAC patients. Besides this, FBXO28 expression was related to cell stemness, tumor size, and pathological grading, but no significant correlation with promoter methylation. Notably, FBXO28 expression was strongly linked to the infiltration of multiple immune cells, such as B cells, CD8^+^ T cells, macrophages, neutrophils, and dendritic cells. The functional analysis revealed that FBXO28 may be involved in protein transport, protein deubiquitination, EGFR signaling pathway, and regulation of actin cytoskeleton.

### FBXO32

The promoter hypermethylation of FBXO32 was responsible for its downregulation in ovarian cancer cells. A profound methylation frequency of FBXO32 was detected in advanced-stage ovarian cancer, which predicted a shorter progression-free survival (48). The re-expression of FBXO32 dampened cell growth in platinum-resistant ovarian cancer as a result of being re-sensitized to cisplatin and increased apoptosis (48). A similar situation of aberrant methylation of FBXO32 could be found in esophageal squamous cell carcinoma (49). Furthermore, FBXO32 inhibited breast cancer oncogenesis and progression *via* interacting with KLF4 for its ubiquitination and degradation, a critical factor for cell fate decisions (50). Consistent with our findings, we also found aberrant promoter methylation of FBXO32, but the discordant result was that FBXO32 was hypomethylated and highly expressed in PanIN and PDAC, in comparison to normal tissues, as well as closely relevant in cell stemness, tumor size, lymphatic metastasis, tumor differentiation, tumor staging, and the prognosis of PDAC patients. Among pancreatic precancerous lesions, FBXO32 had the highest expression in IPMC. Furthermore, FBXO32 expression is also related to immune infiltration, *e*.*g*., CD8^+^ T cells, NK cells, macrophages, neutrophils, and dendritic cells. The functional analysis demonstrated that FBXO32 may be involved in cell differentiation, angiogenesis, regulation of actin cytoskeleton, and PI3K-Akt signaling pathway, which remains to be further explored.

### FBXO45

FBXO45 propelled ubiquitin-mediated proteolysis of the tumor-suppressor PAR4 to control cancer cell survival (51). Specifically, E3 ubiquitin ligase FBXO45 could interact with PAR4, a PRKC apoptosis WT1 regulator, *via* a short consensus sequence motif in the cytoplasm to influence its ubiquitylation and proteasomal degradation, which, in turn, governs cell apoptosis (51). Furthermore, FBXO45 modulated the process of EMT *via* mediating the ubiquitination and degradation of substantial EMT-related transcription factors, such as Twist1, Snai1/2, and Zeb1/2, in tumor cells (52). In gastric cancer, the expression levels of FBXO45 in tumor tissues were increased compared with those in normal tissues. Unexpectedly, patients with high FBXO45 expression had longer survival than those with low expression (53). Herein we found that FBXO45 expression was markedly upregulated in PDAC tissues in both mRNA and protein levels and was inversely relevant in the OS and DFS of PDAC patients based on multiple online databases. FBXO45 expression was related to the degree of tumor differentiation, tumor staging, and P53 mutation in PDAC. The expression level of FBXO45 was higher in IPMC than in normal pancreatic tissues. Additionally, the expression levels of FBXO45 were related to immune infiltration, *i*.*e*., B cells, CD8^+^ T cells, CD4^+^ T cells, NK cells, macrophages, neutrophils, and dendritic cells. The functional analysis showed that FBXO45 was involved in ErbB signaling pathway and T cell receptor signaling pathway.

Among six-FBXOs, FBXO32 had attracted our attention since it exhibited a strong correlation with all the clinicopathological parameters investigated as detailed above. In terms of prognosis, FBXO32 expression could well predict the clinical outcomes of PDAC patients in both OS and DFS. For diagnosis, FBXO32 was tightly associated with clinicopathologic characteristics, cell stemness, and immune infiltration. Besides this, we noticed that FBXO32 was not only significantly upregulated in PDAC tissues but also highly expressed in some precancerous tissues, such as IPMC and PanIN, which provided a strong support for the early diagnosis of PDAC. Regarding treatment, we speculated that FBXO32 was likely to play a role in PDAC initiation and progression as well as reprogramming of the tumor immune microenvironment through regulating cell differentiation, angiogenesis, actin cytoskeleton, and some important signaling pathways, such as PI3K/Akt signaling pathway. By developing therapeutic agents targeting FBXO32, it is possible to control and block the development and malignant transformation of PDAC. Therefore, FBXO32 might be a promising prognostic/diagnostic/therapeutic target for PDAC.

## Conclusion

In the present study, we identified that FBXO1, FBXO20, FBXO22, FBXO28, FBXO32, and FBXO45 are highly expressed in PDAC tissues, which are potential unfavorable prognostic factors for PDAC patients. The six FBXOs are strongly associated with clinicopathological features, promoter methylation, immune infiltration, and genetic mutation in PDAC based on different public databases. So far, there is a paucity in the literature studying the roles of six-FBXOs in PDAC. Therefore, the specific roles and underlying mechanisms of six-FBXOs in PDAC are worth further investigation through a large number of related experiments *in vitro* and *in vivo*. To sum up, our study indicates that six-FBXOs might act an oncogenic role to promote PDAC progression and serve as the potential targets for PDAC diagnosis and treatment.

## Data Availability Statement

The datasets presented in this study can be found in online repositories. The names of the repository/repositories and accession number(s) can be found in the article/**Supplementary Material**.

## Ethics Statement

The study has been approved by the Ethics Committee of Peking Union Medical College Hospital. Since all the data were collected and downloaded from the public online databases, it was certain that all written informed consent had already been obtained.

## Author Contributions

YZ conceived this study, analyzed the data, and drafted the manuscript. QLiu reviewed and revised the manuscript. MC, MW, SH, and JG collected the data and reviewed the manuscript. QLiao was responsible for project administration and supervision. All authors contributed to the article and approved the submitted version.

## Funding

This work was supported by the National Natural Science Foundation of China (82172765, 81872501, 81673023, 81272573, and 81502068), Beijing Natural Science Foundation (7172177), CAMS Innovation Fund for Medical Sciences (CIFMS, 2021-I2M-1-002) and Youth Foundation of Peking Union Medical College Hospital (pumch201911866).

## Conflict of Interest

The authors declare that the research was conducted in the absence of any commercial or financial relationships that could be construed as a potential conflict of interest.

## Publisher’s Note

All claims expressed in this article are solely those of the authors and do not necessarily represent those of their affiliated organizations, or those of the publisher, the editors and the reviewers. Any product that may be evaluated in this article, or claim that may be made by its manufacturer, is not guaranteed or endorsed by the publisher.

## Abbreviations

ANCT, adjacent non-cancerous tissues; BP, biological process; CC, cellular component; CCNF, cyclin F; CI, confidence interval; DFS, disease-free survival; EMT, epithelial–mesenchymal transition; GO, gene ontology; hn-PDEC, human normal pancreatic ductal epithelial cells; HPA, Human Protein Atlas; HR, hazard ratio; IHC, immunohistochemistry; IPMA, intraductal papillary-mucinous adenoma; IPMC, intraductal papillary-mucinous carcinoma; IPMN, intraductal papillary-mucinous neoplasm; KEGG, Kyoto Encyclopedia of Genes and Genomes; LMO7, LIM (Lin11, Isl-1, and Mec-3) domain 7; MF, molecular function; OS, overall survival; PAAD, pancreatic adenocarcinoma; PanIN, pancreatic duct intraepithelial neoplasia; PDAC, pancreatic ductal adenocarcinoma; RRM2, ribonucleotide reductase M2; SCF, SKP1-cullin 1-F-box.
