# Supplementary material for: Comprehensive Analysis of Expression, Prognostic Value, and Immune Infiltration for Ubiquitination-Related FBXOs in Pancreatic Ductal Adenocarcinoma
Source: Front Immunol. 2022 Jan 3;12:774435. doi: 10.3389/fimmu.2021.774435 (PMC8761623; doi:10.3389/fimmu.2021.774435)
Supplement: Supplementary file 1 [file DataSheet_1.pdf]

Supplementary Material

1 Supplementary Figures

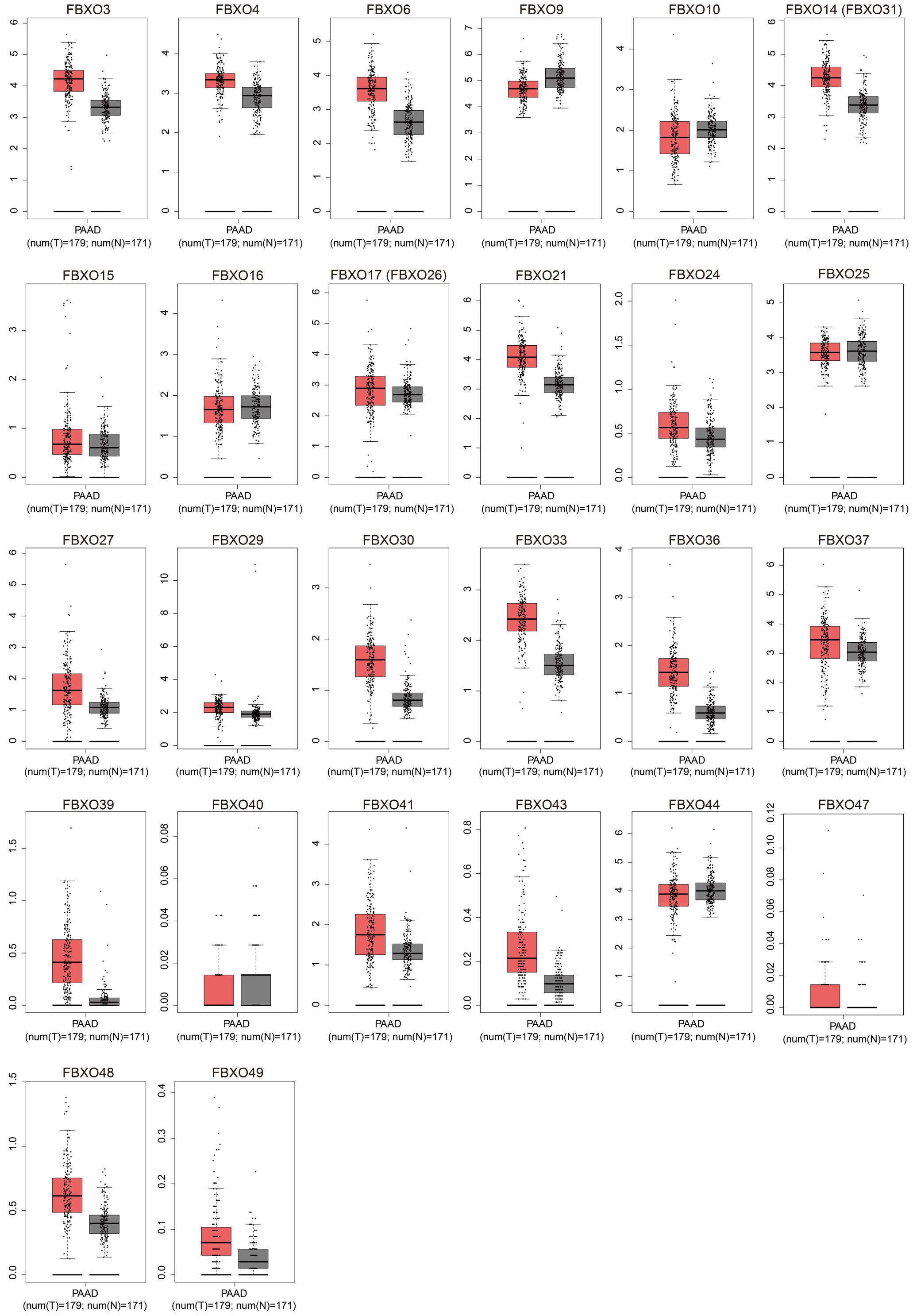

Supplementary Figure 1. FBXO family members with no difference in expression between PDAC (n=179) and normal pancreatic tissues (n=171) in the GEPIA based on TCGA and GTEx database.

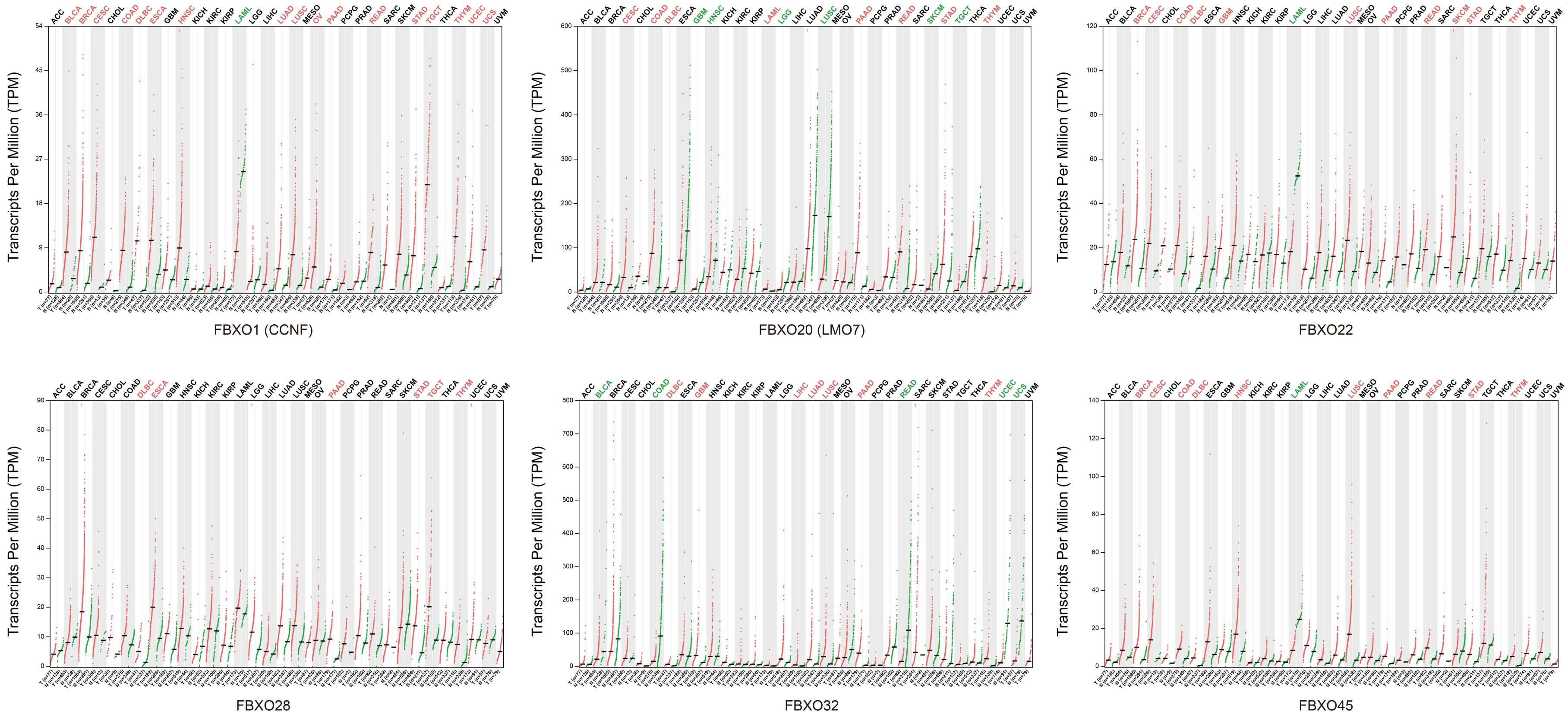

Supplementary Figure 2. Transcriptional levels of six-FBXOs in 33 types of tumor and corresponding normal tissues. PAAD, pancreatic adenocarcinoma. Green words refer to low expression in tumor tissues and red words refer to high expression.

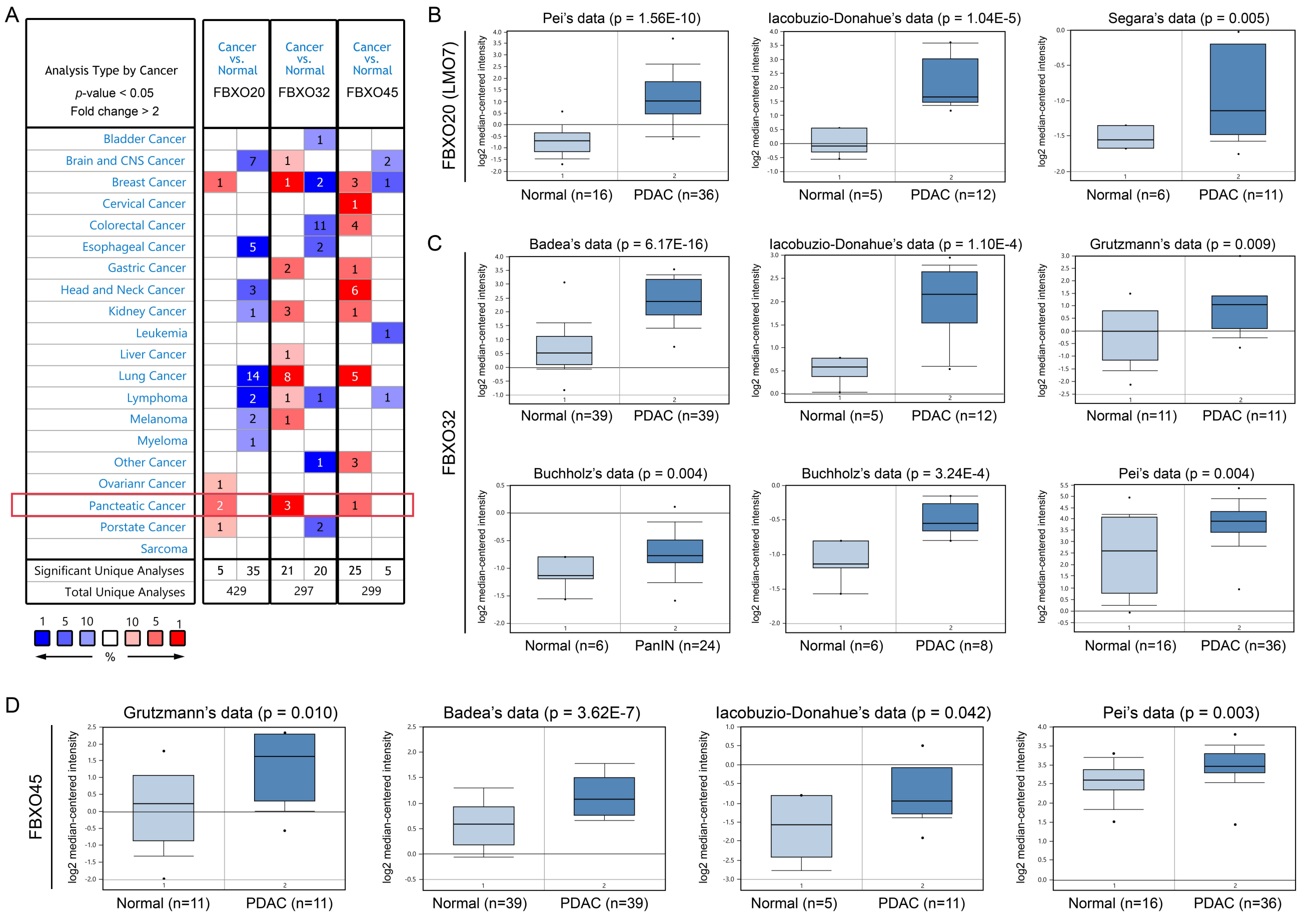

Supplementary Figure 3. The expression levels of FBXO20, FBXO32, and FBXO45 were significantly increased in PDAC tissues (Oncomine). **(A)** The abnormal expression of FBXO20, 32, and 45 in a variety of human tumors (Fold chang > 2, *p*-value < 0.05). **(B)** The mRNA levels of FBXO20 (LMO7) were shown in three different PDAC datasets. **(C)** The transcriptional levels of FBXO32 were analyzed in five PDAC datasets and one PanIN dataset. **(D)** The expression levels of FBXO45 in four different PDAC datasets.

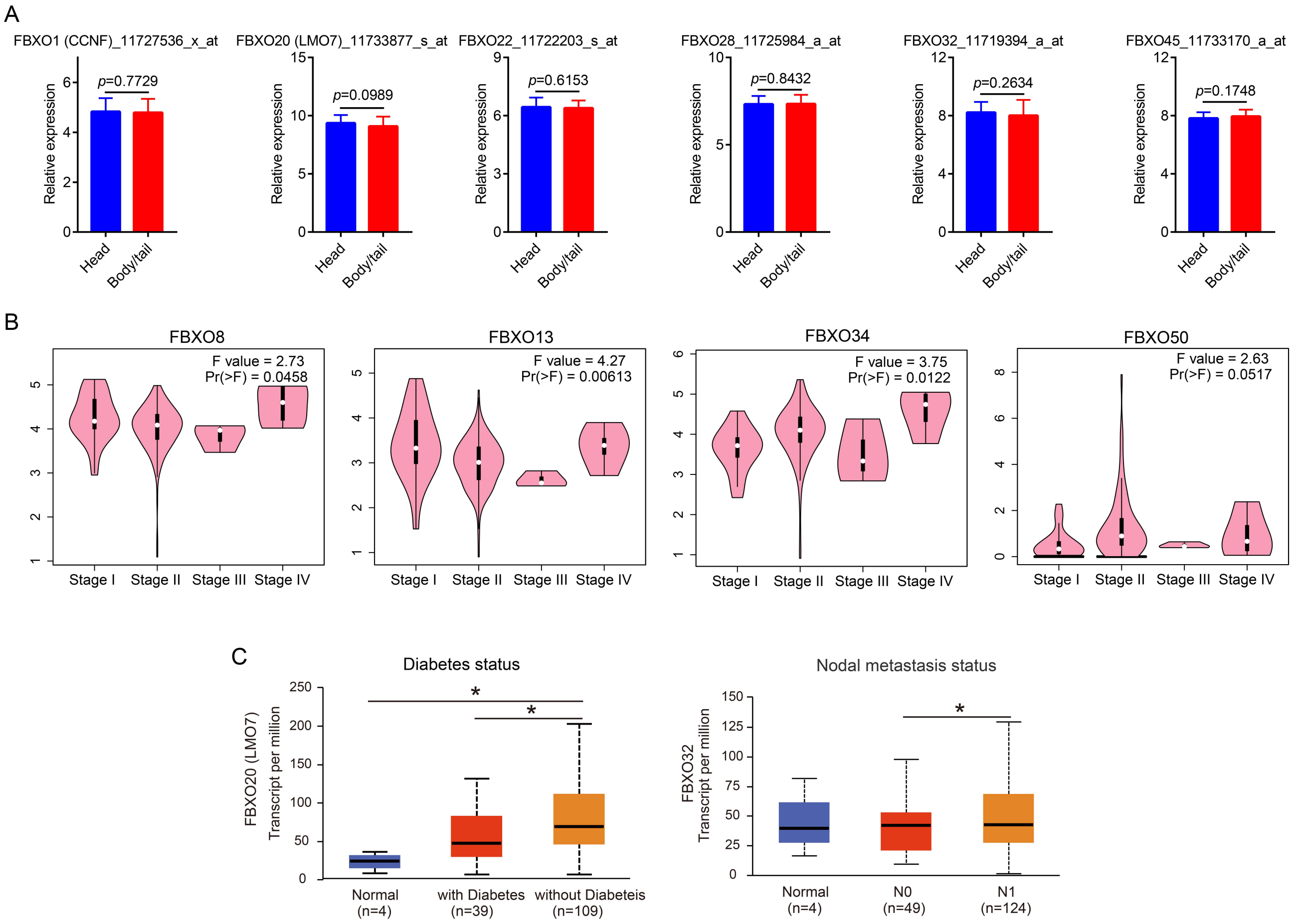

Supplementary Figure 4. Correlation analysis of the six-FBXOs and clinicopathological characteristics of PDAC patients. **(A)** The comparation between head (n=93) and body/tail (n=25) of pancreatic cancer from GSE62165. The code behind the gene is the probe name. (**B)** The expression of FBXO family members in different pathological stages of PDAC from GEPIA. **(C)** The expression of FBXO20 (LMO7) in PDAC patients with/without diabetes and the mRNA level of FBXO32 in PDAC patients with/with nodal metastasis using UALCAN database. *$P < 0.05$.

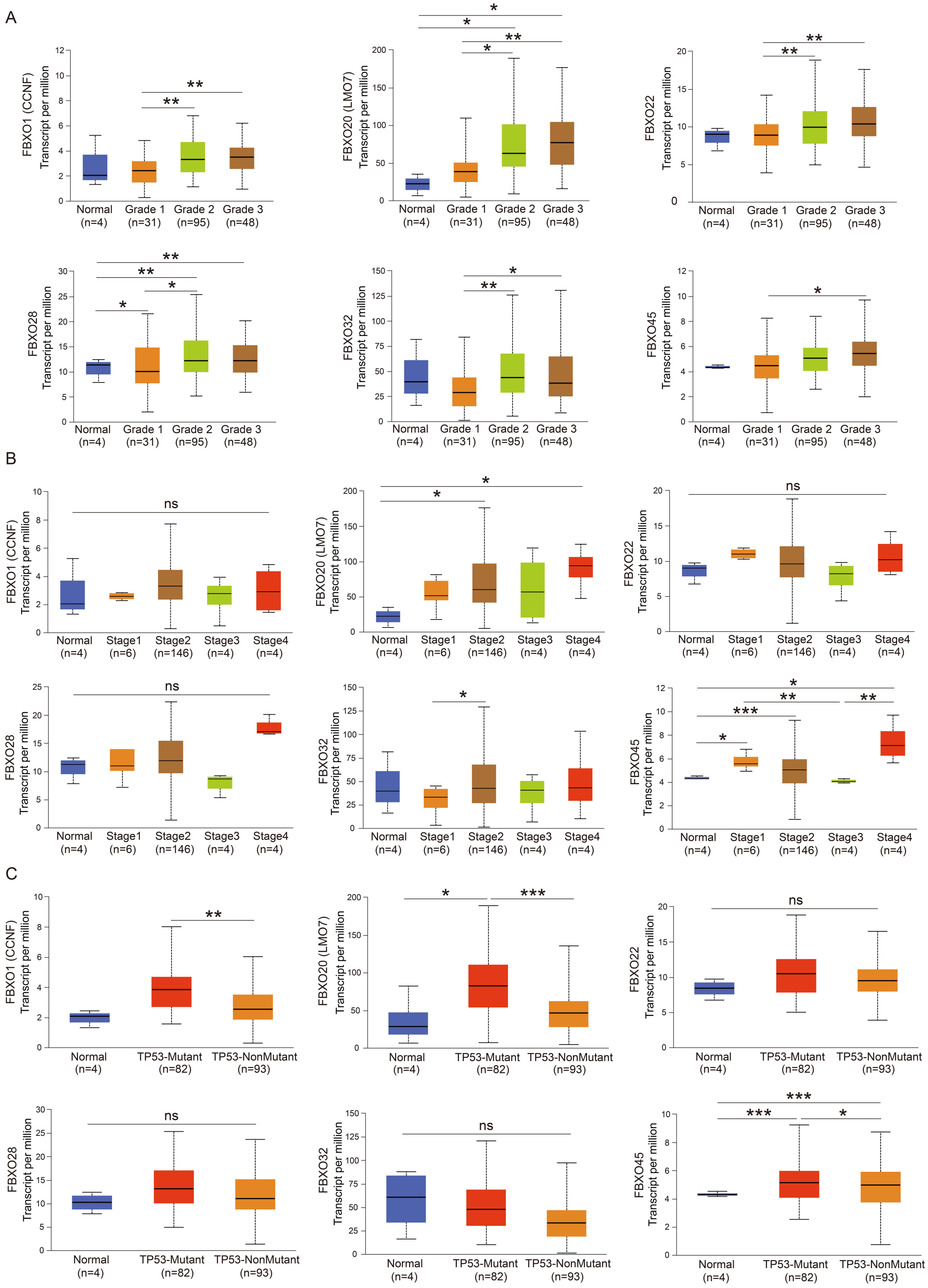

Supplementary Figure 5. Correlation between six-FBXOs expression and clinicopathological characteristics, as well as P53 mutation in PDAC using UALCAN database. **(A)** The transcriptional levels of six-FBXOs in different differentiation degrees of PDAC. **(B)** The expression levels of six-FBXOs in different pathological stages. **(C)** The expression levels of six-FBXOs in PDAC patients with/without P53 mutation. **P* < 0.05, ***P* < 0.01, ****P* < 0.001, *****P* < 0.0001. n.s., not significant difference.

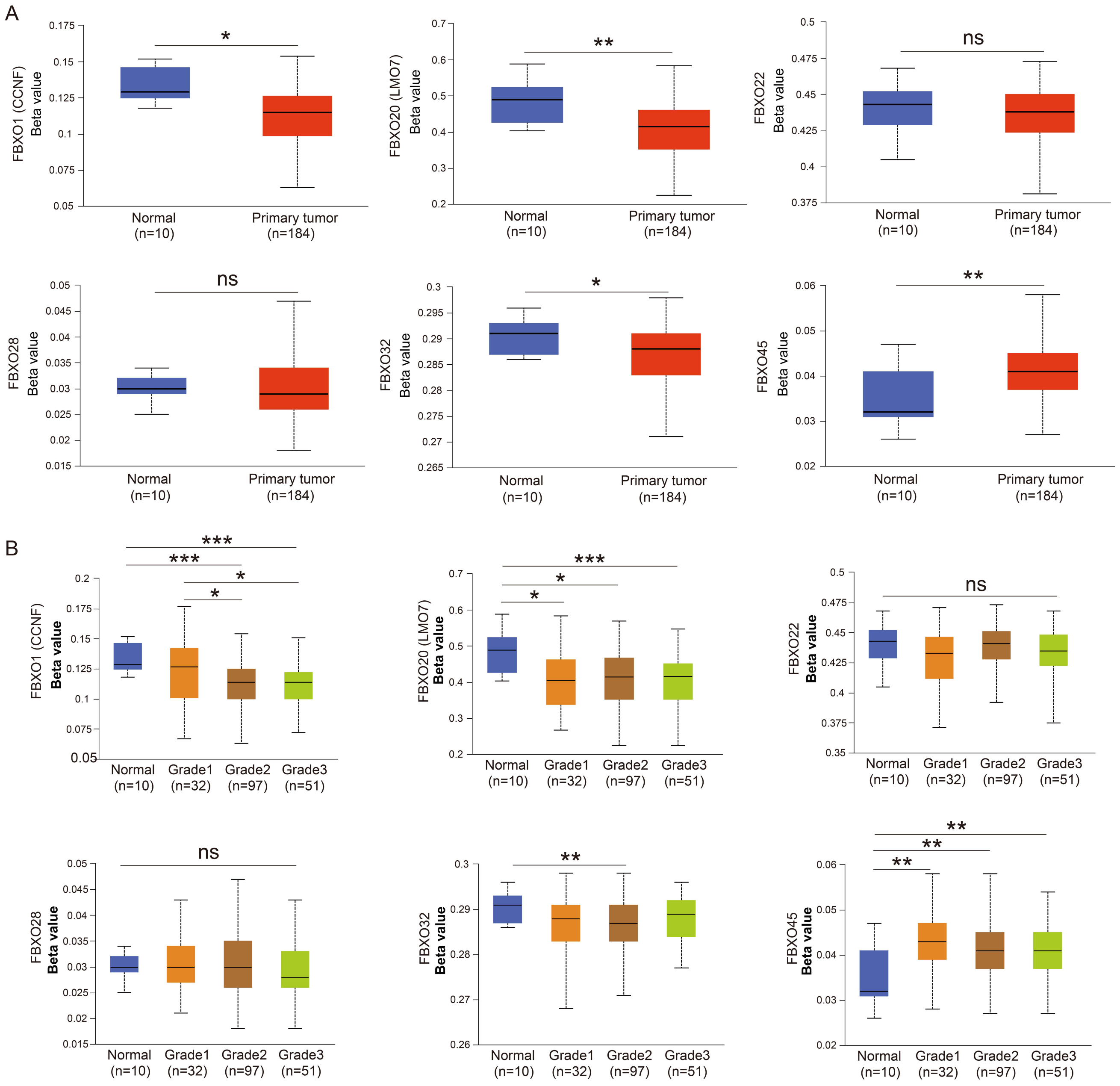

Supplementary Figure 6. Methylation degree of the six-FBXOs in PDAC and normal pancreatic tissues, and different pathological differentiation using UALCAN database. **(A)** Methylation levels of the six-FBXOs in PDAC and normal tissues. **(B)** Methylation levels of the six-FBXOs in different degrees of differentiation in PDAC. **P* < 0.05, ***P* < 0.01, ****P* < 0.001; n.s., not significant difference.

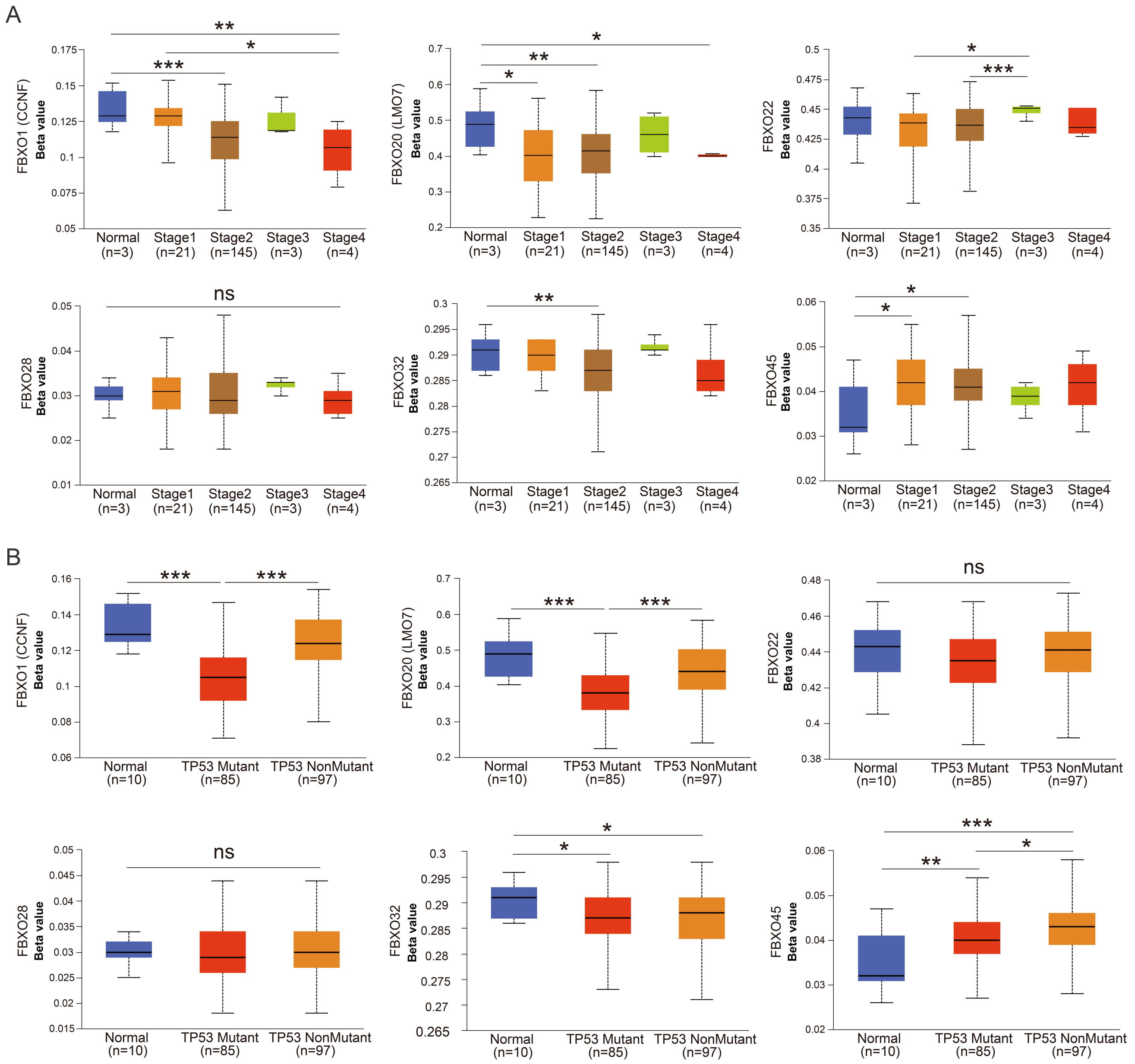

Supplementary Figure 7. Methylation degree of the six-FBXOs in different tumor staging and in PDAC patients with/without P53 mutation using UALCAN database. **(A)** Methylation levels of the six-FBXOs in different tumor staging of PDAC patients. **(B)** Methylation levels of the six-FBXOs in PDAC patients with/without P53 mutation. *$P < 0.05$, **$P < 0.01$, ***$P < 0.001$; n.s., not significant difference.

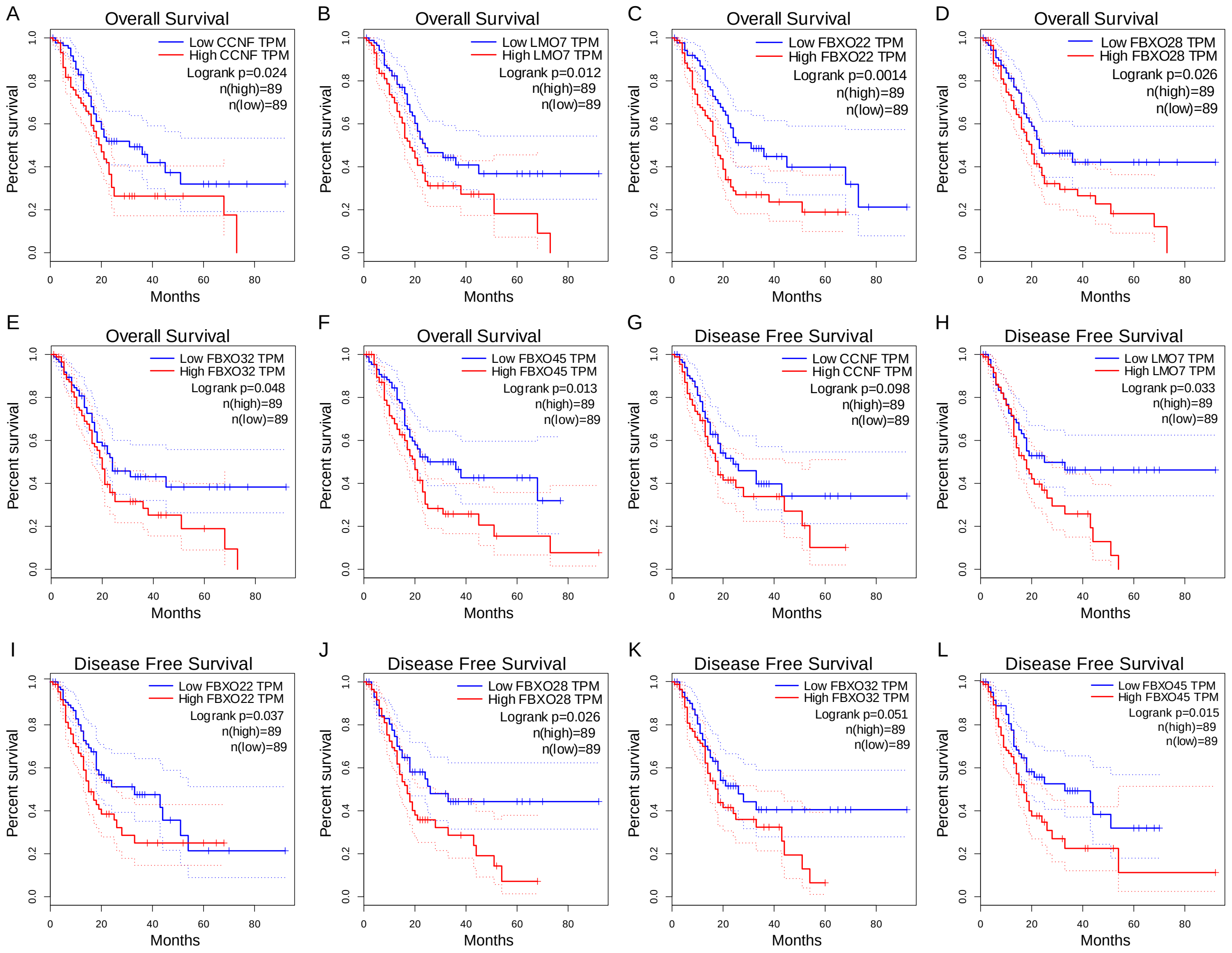

Supplementary Figure 8. The prognostic analysis of the six-FBXOs in PDAC using GEPIA database. **(A-F)** The overall survival of the six-FBXOs in PDAC patients. **(G-L)** The disease-free survival of the six-FBXOs in PDAC patients. “Median” as the “Group Cutoff” (89 vs 89).

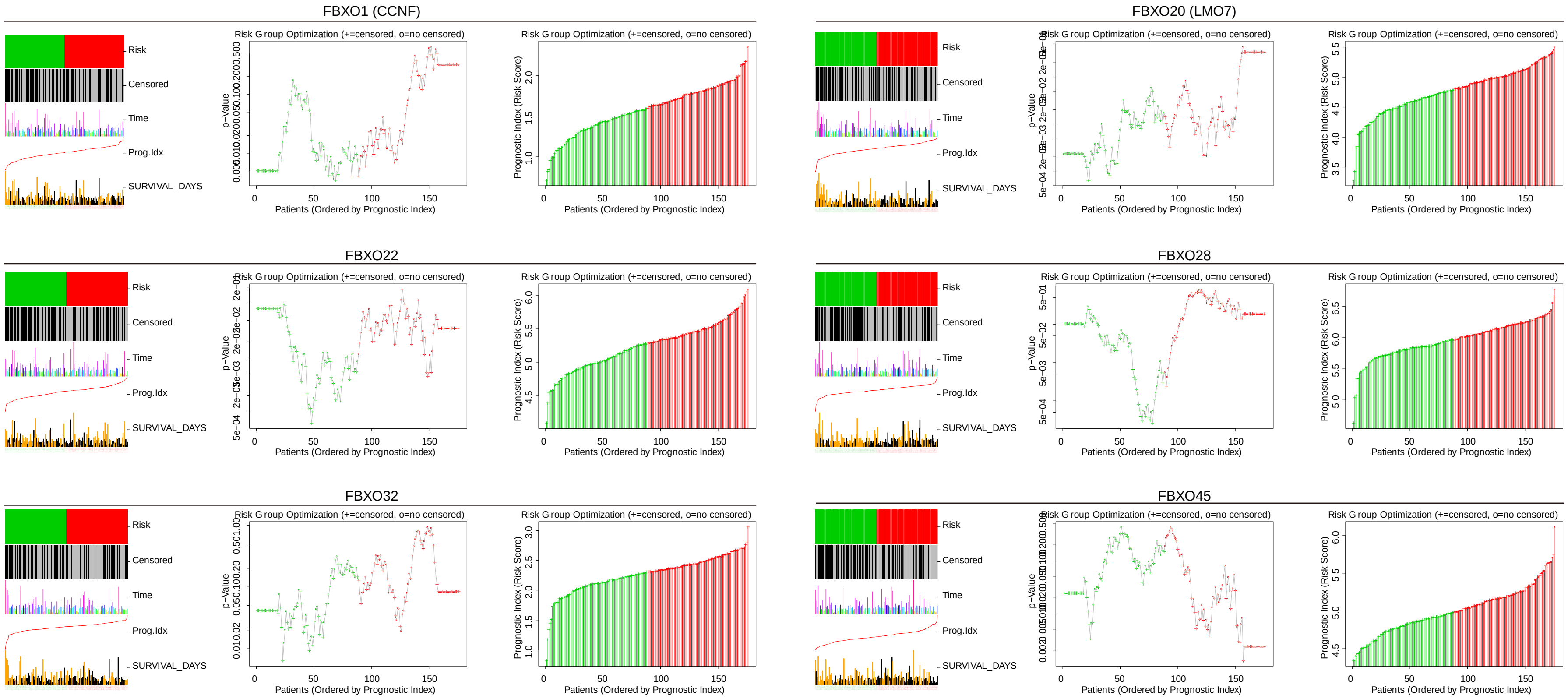

Supplementary Figure 9. The prognostic index, risk group optimization, and censored/non- censored status of the six-FBXOs in PDAC cohort using the SurvExpress database.

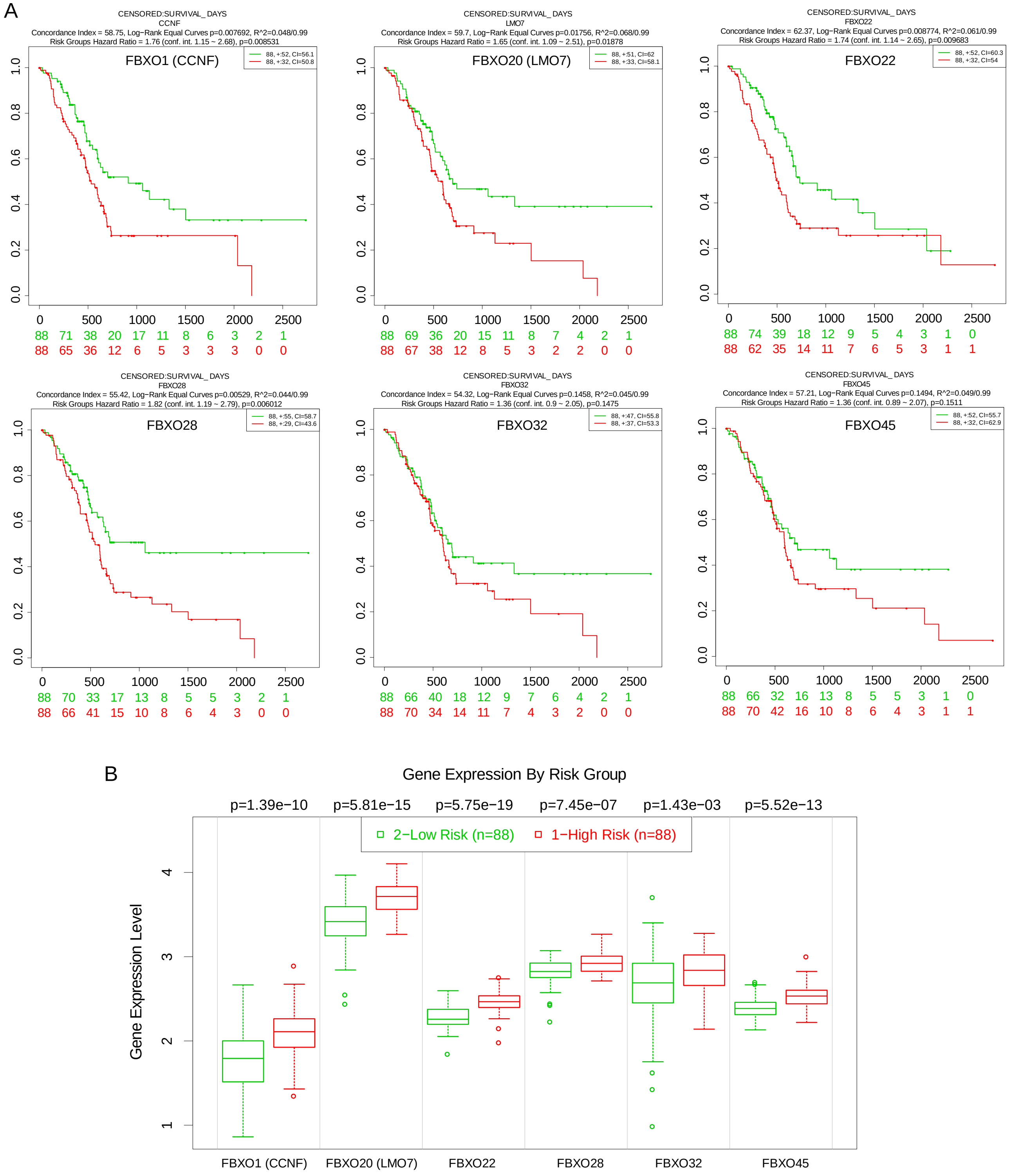

Supplementary Figure 10. The prognostic evaluation **(A)** of the six-FBXOs in PDAC patients (N=176) and the expression levels of six-FBXOs in low-risk group and high-risk group **(B)** according PDAC patient's survival time using SurvExpress database.

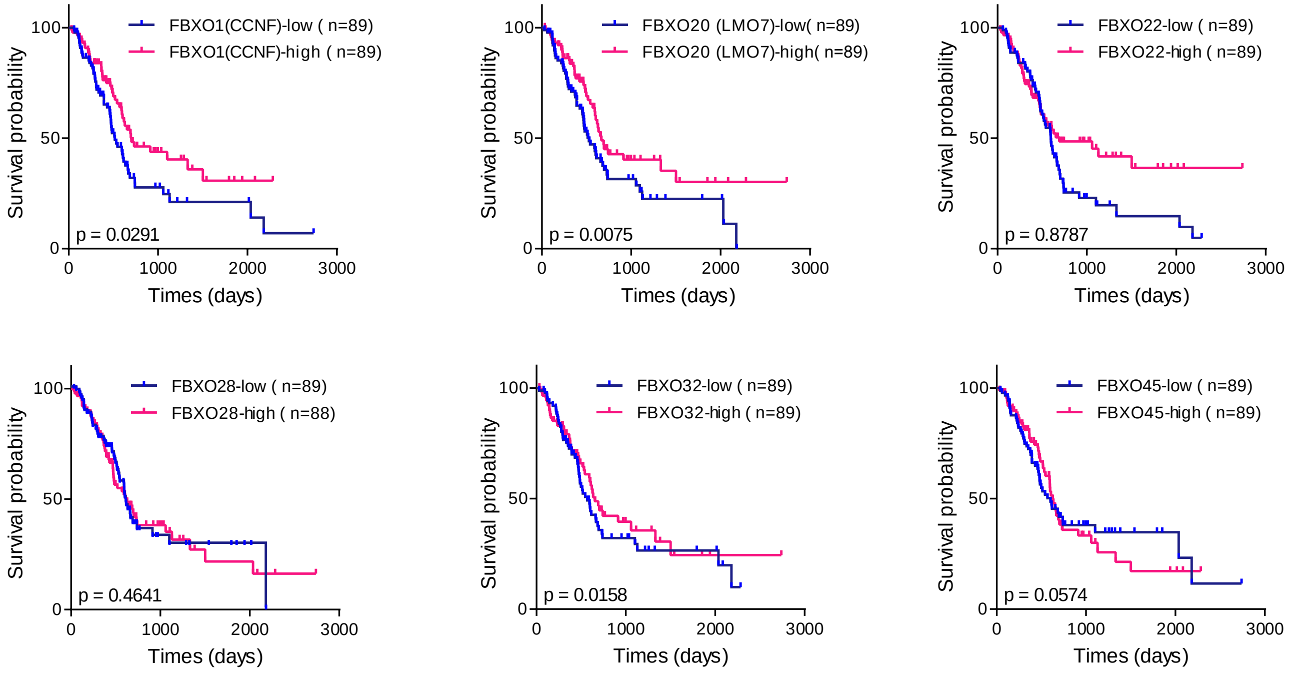

Supplementary Figure 11. The prognostic analysis of the six-FBXOs in hypermethylated group and hypomethylated group of PDAC patients using LinkedOmics database.

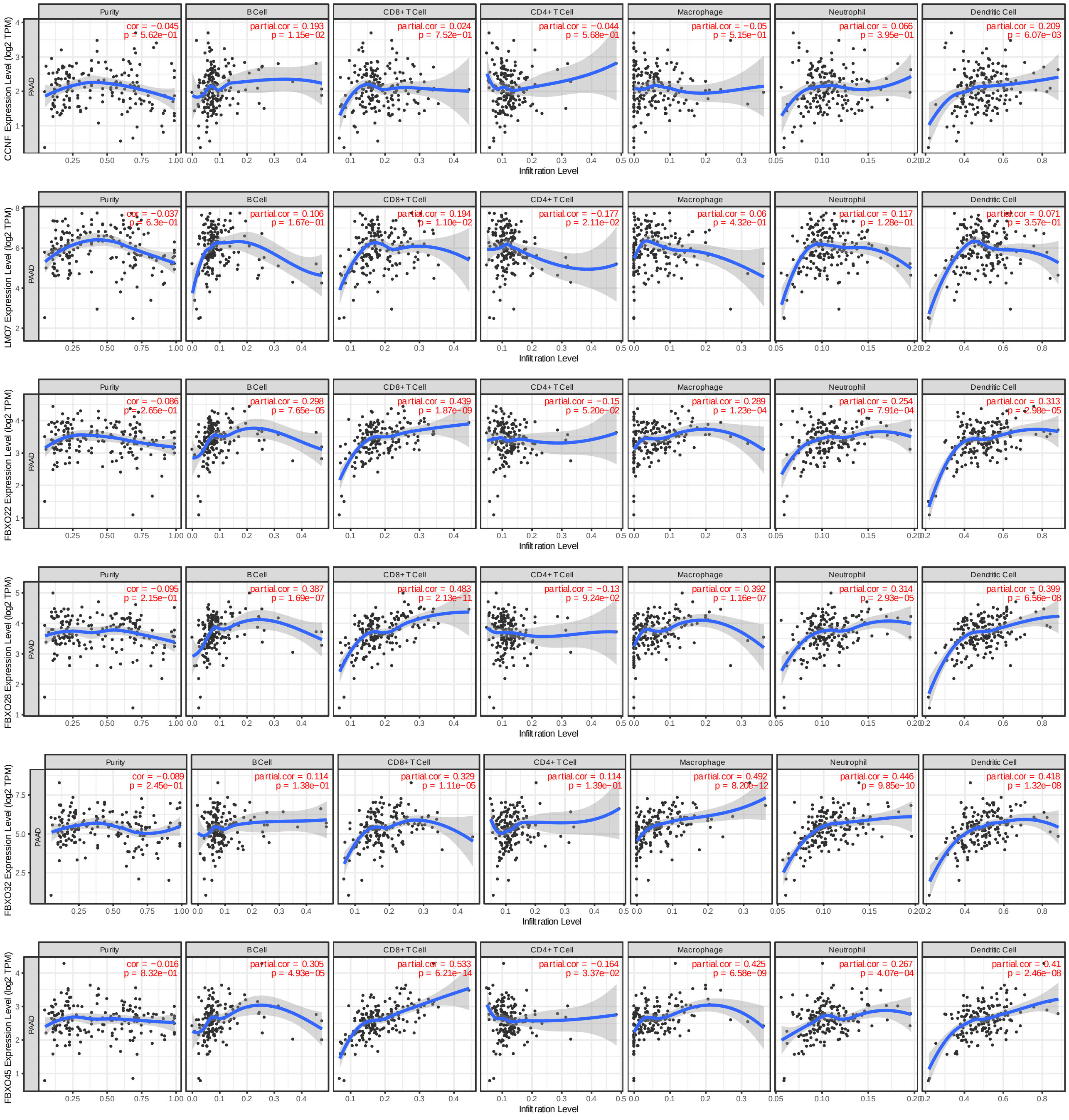

Supplementary Figure 12. Correlation of mRNA expression of six-FBXOs with immune infiltration level in PDAC (TIMER, Spearman correlation), including B cells, $CD8^+$ T cells, $CD4^+$ T cells, macrophages, neutrophils, and dendritic cells.

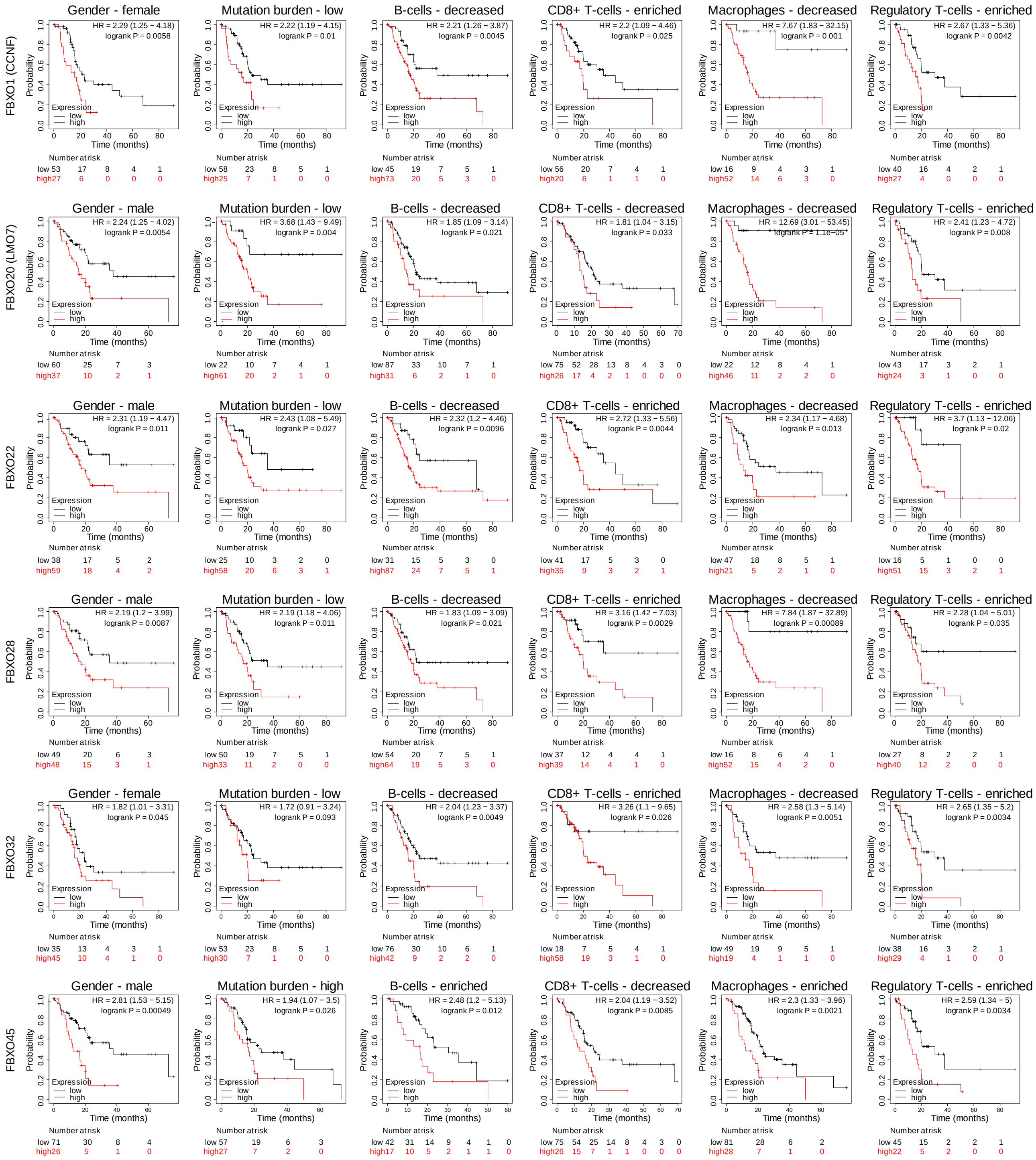

Supplementary Figure 13. The subgroup analysis of overall survival of the six-FBXOs in gender, mutation burden, and immune cell infiltration, including B cells, CD8$^+$ T cells, macrophages, and regulatory T cells, in PDAC patients using the Kaplan Meier-plotter. “Auto select best cutoff” was selected. NA, not available.

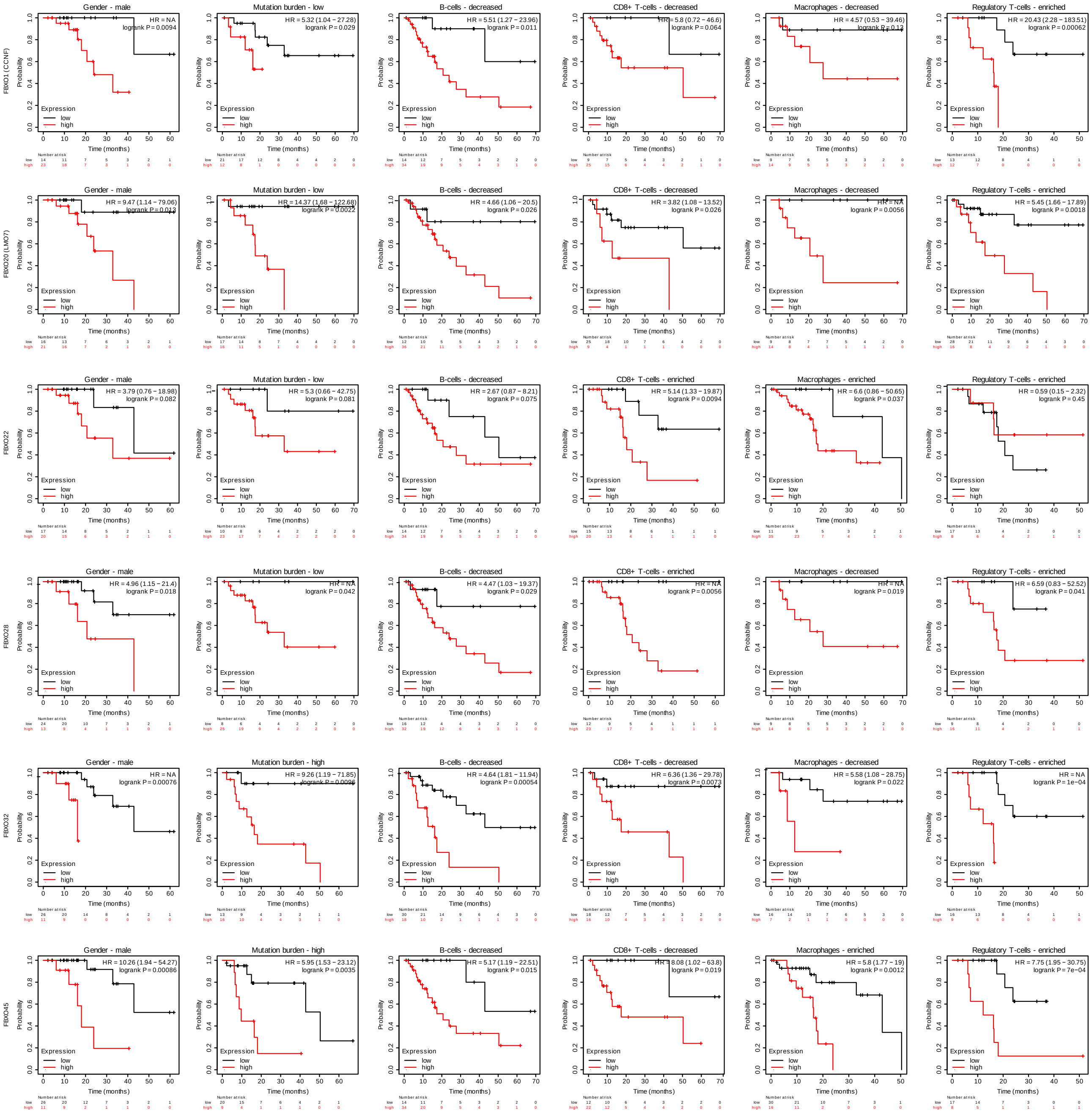

Supplementary Figure 14. The subgroup analysis of DFS of the six-FBXOs in gender, mutation burden, and immune cell infiltration, including B cells, CD8^{+} T cells, macrophages, and regulatory T cells, in PDAC patients using the Kaplan Meier-plotter. "Auto select best cutoff" was selected. NA, not available.

2 Supplementary Tables

Supplementary Table 1. The primer sequences of real-time quantitative PCR

Genes	Primer sequences
FBXO1-F	5′-ATGGCTCACGGACAACACTT-3′
FBXO1-R	5′-TGGGGACTCGAATCTTCCCT-3′
FBXO20-F	5′-AATCAGCATAAACCAGACGCC-3′
FBXO20-R	5′-CTGGGCTACCTGCTTCAACT-3′
FBXO22-F	5′-TAAGGTGGGAGCCAGTAA-3′
FBXO22-R	5′-AAGAAGCCGAATAAGGGA-3′
FBXO28-F	5′-ATGTCAATTCTACCAGAGCCCC-3′
FBXO28-R	5′-AGCTGCATTGTTGTTAGGGC-3′
FBXO32-F	5′-ATTGGTTAGTGATAGTTAAGGGT-3′
FBXO32-R	5′-GGGATAACGGTGTTTTGG-3′
FBXO45-F	5′-AGTGCCAAGGTTATGTGGCATTGCTG-3′
FBXO45-R	5′-AGAAAGCCACTGTCATCCGTCCAAAG-3′
GAPDH-F	5′-CATGAGAAGTATGACAACAGCCT-3′
GAPDH-R	5′-AGTCCTTCCACGATACCAAAGT-3′

F, forward; R, reverse.

Supplementary Table 2. The prognostic subgroup analysis of six-FBXOs from Kaplan Meier-plotter in PDAC

Genes	OS			RFS		
	Subgroups	HR (95% CI)	*p*-value	Subgroups	HR (95% CI)	*p*-value
FBXO1 (CCNF)	Gender - female	2.29 (1.25-4.18)	0.0058	Gender - male	NA	0.0094
	Mutation burden - low	2.22 (1.19 − 4.15)	0.0105	Mutation burden - low	5.32 (1.04 − 27.28)	0.0295
	B-cells - decreased	2.21 (1.26 − 3.87)	0.0045	B-cells - decreased	5.51 (1.27 − 23.96)	0.0107
	$CD8^+$ T cells - enriched	2.2 (1.09 − 4.46)	0.0249	$CD8^+$ T cells - decreased	5.8 (0.72 − 46.6)	0.0639
	Macrophages - decreased	7.67 (1.83 − 32.15)	0.0010	Macrophages - decreased	4.57 (0.53 − 39.46)	0.1296
	Regulatory T cells - enriched	2.67 (1.33 − 5.36)	0.0042	Regulatory T cells - enriched	20.43 (2.28 − 183.51)	0.0006
FBXO20 (LMO7)	Gender - male	2.24 (1.25 − 4.02)	0.0054	Gender - male	9.47 (1.14 − 79.06)	0.0127
	Mutation burden - low	3.68 (1.43 − 9.49)	0.0040	Mutation burden - low	14.37 (1.68 − 122.68)	0.0022
	B-cells - decreased	1.85 (1.09 − 3.14)	0.0207	B-cells - decreased	4.66 (1.06 − 20.5)	0.0262
	$CD8^+$ T cells - decreased	1.81 (1.04 − 3.15)	0.0333	$CD8^+$ T cells - decreased	3.82 (1.08 − 13.52)	0.0261
	Macrophages - decreased	12.69 (3.01 − 53.45)	1.10E-05	Macrophages - decreased	NA	0.0056
	Regulatory T cells - enriched	2.41 (1.23 − 4.72)	0.0080	Regulatory T cells - enriched	5.45 (1.66 − 17.89)	0.0018
FBXO22	Gender - male	2.31 (1.19 − 4.47)	0.0105	Gender - male	3.79 (0.76 − 18.98)	0.0824
	Mutation burden - low	2.43 (1.08 − 5.49)	0.0273	Mutation burden - low	5.3 (0.66 − 42.75)	0.0808
	$CD8^+$ T cells - enriched	2.72 (1.33 − 5.56)	0.0044	$CD8^+$ T cells - enriched	5.14 (1.33 − 19.87)	0.0094
	B-cells - decreased	2.32 (1.2 − 4.46)	0.0096	B-cells - decreased	2.67 (0.87 − 8.21)	0.0746
	Macrophages - decreased	2.34 (1.17 − 4.68)	0.0132	Macrophages - enriched	6.6 (0.86 − 50.65)	0.0368
	Regulatory T cells - enriched	3.7 (1.13 − 12.06)	0.0201	Regulatory T cells - enriched	0.59 (0.15 − 2.32)	0.4471
FBXO28	Gender - male	2.19 (1.2 − 3.99)	0.0087	Gender - male	4.96 (1.15 − 21.4)	0.0181
	Mutation burden - low	2.19 (1.18 − 4.06)	0.0110	Mutation burden - low	NA	0.0424
	B-cells - decreased	1.83 (1.09 − 3.09)	0.0208	B-cells - decreased	4.47 (1.03 − 19.37)	0.0286
	$CD8^+$ T cells - enriched	3.16 (1.42 − 7.03)	0.0029	$CD8^+$ T cells - enriched	NA	0.0056
	Macrophages - decreased	NA	0.0009	Macrophages - decreased	NA	0.0185
	Regulatory T cells - enriched	2.28 (1.04 − 5.01)	0.0346	Regulatory T cells - enriched	6.59 (0.83 − 52.52)	0.0406
FBXO32	Gender - female	1.82 (1.01 − 3.31)	0.0447	Gender - male	NA	0.0008
	Mutation burden - low	1.72 (0.91 − 3.24)	0.0927	Mutation burden - high	9.26 (1.19 − 71.85)	0.0096
	B-cells - decreased	2.04 (1.23 − 3.37)	0.0049	B-cells - decreased	4.64 (1.81 − 11.94)	0.0005
	$CD8^+$ T cells - enriched	3.26 (1.1 − 9.65)	0.0260	$CD8^+$ T cells - decreased	6.36 (1.36 − 29.78)	0.0073
	Macrophages - decreased	2.58 (1.3 − 5.14)	0.0051	Macrophages - decreased	5.58 (1.08 − 28.75)	0.0220
	Regulatory T cells - enriched	2.65 (1.35 − 5.2)	0.0034	Regulatory T cells - enriched	NA	0.0001

FBXO45	Gender - male	2.81 (1.53 − 5.15)	0.0005	Gender - male	10.26 (1.94 − 54.27)	0.0009
	Mutation burden - high	1.94 (1.07 − 3.5)	0.0258	Mutation burden - high	5.95 (1.53 − 23.12)	0.0035
	B-cells - enriched	2.48 (1.2 − 5.13)	0.0116	B-cells - decreased	5.17 (1.19 − 22.51)	0.0150
	$CD8^+$ T cells - decreased	2.04 (1.19 − 3.52)	0.0085	$CD8^+$ T cells - decreased	8.08 (1.02 − 63.8)	0.0190
	Macrophages - enriched	2.3 (1.33 − 3.96)	0.0021	Macrophages - enriched	5.8 (1.77 − 19)	0.0012
	Regulatory T cells - enriched	2.59 (1.34 − 5)	0.0034	Regulatory T cells - enriched	7.75 (1.95 − 30.75)	0.0007

HR, hazard ratio; CI, confidence interval; NA, not available.

Supplementary Table 3. Prognostic analysis of six-FBXOs between altered group and unaltered group from cBioportal in PDAC

Prognosis types	Mutation Group	Number of Cases (Total)	Number of Events	Median Months Overall (95% CI)	*p*-value
Overall	Altered group	68	44	16.80 (12.53 - 22.72)	**0.0331**
	Unaltered group	100	45	22.85 (19.96 - 49.38)	
Progression Free	Altered group	68	48	13.05 (9.60 - 17.92)	**0.0428**
	Unaltered group	100	51	16.87 (12.92 - 32.45)	
Disease-specific	Altered group	64	35	19.50 (15.98 - NA)	0.0592
	Unaltered group	98	35	24.07 (20.61 - NA)	
Disease Free	Altered group	22	10	23.54 (16.18 - NA)	0.2020
	Unaltered group	41	12	49.68 (27.32 - NA)	

CI, confidence interval.

Supplementary Table 4. The top 20 genes between FBXO family altered group and unaltered group in PDAC

Gene	Cytoband	Altered group	Unaltered group	Log Ratio	Enriched in	*q*-Value	*p*-Value
ADCY8	8q24.22	21 (30.88%)	0 (0.00%)	>10	Altered group	3.92E-06	6.56E-10
ASAP1	8q24.21-q24.22	20 (29.41%)	0 (0.00%)	>10	Altered group	3.92E-06	2.02E-09
EFR3A	8q24.22	20 (29.41%)	0 (0.00%)	>10	Altered group	3.92E-06	2.02E-09
KCNQ3	8q24.22	20 (29.41%)	0 (0.00%)	>10	Altered group	3.92E-06	2.02E-09
PCAT1	8q24.21	20 (29.41%)	0 (0.00%)	>10	Altered group	3.92E-06	2.02E-09
SQLE	8q24.13	20 (29.41%)	0 (0.00%)	>10	Altered group	3.92E-06	2.02E-09
WASHC5	8q24.13	20 (29.41%)	0 (0.00%)	>10	Altered group	3.92E-06	2.02E-09
ASAP1-IT1	8q24.21	19 (27.94%)	0 (0.00%)	>10	Altered group	3.92E-06	6.15E-09
ASAP1-IT2	8q24.21	19 (27.94%)	0 (0.00%)	>10	Altered group	3.92E-06	6.15E-09
C8ORF76	8q24.13	19 (27.94%)	0 (0.00%)	>10	Altered group	3.92E-06	6.15E-09
DERL1	8q24.13	19 (27.94%)	0 (0.00%)	>10	Altered group	3.92E-06	6.15E-09
DNAAF11	8q24.22	19 (27.94%)	0 (0.00%)	>10	Altered group	3.92E-06	6.15E-09
FAM83A	8q24.13	19 (27.94%)	0 (0.00%)	>10	Altered group	3.92E-06	6.15E-09
FAM91A1	8q24.13	19 (27.94%)	0 (0.00%)	>10	Altered group	3.92E-06	6.15E-09
FER1L6	8q24.13	19 (27.94%)	0 (0.00%)	>10	Altered group	3.92E-06	6.15E-09
LINC00861	8q24.13	19 (27.94%)	0 (0.00%)	>10	Altered group	3.92E-06	6.15E-09
LINC00977	8q24.21	19 (27.94%)	0 (0.00%)	>10	Altered group	3.92E-06	6.15E-09
LRATD2	8q24.21	19 (27.94%)	0 (0.00%)	>10	Altered group	3.92E-06	6.15E-09
MTSS1	8q24.13	19 (27.94%)	0 (0.00%)	>10	Altered group	3.92E-06	6.15E-09
